# A secreted *Leishmania* metalloprotease manipulates host iron regulation by targeting the DICER1–miRNA pathway

**DOI:** 10.1016/j.jbc.2025.110851

**Published:** 2025-10-22

**Authors:** Suman Samanta, Sourav Banerjee, Rupak Datta

**Affiliations:** Department of Biological Sciences, Indian Institute of Science Education and Research (IISER) Kolkata, Mohanpur, West Bengal, India

**Keywords:** *leishmania*, GP63, Nramp1, SLC11A1, DICER1, miR-122, iron

## Abstract

Micronutrient sequestration is a powerful host defense mechanism against intracellular pathogens. A key player in this is Nramp1, which effluxes iron from phagolysosomes thereby depriving the engulfed pathogens of this essential element. *Leishmania major* counters this by triggering hepcidin-mediated proteasomal degradation of Nramp1. Interestingly, *L. major* conditioned media induced hepcidin expression and Nramp1 degradation even in uninfected macrophages, resulting in enhanced endo/lysosomal iron levels. This finding suggested that a parasite-derived secretory factor was driving the effect, ultimately leading to the identification of the *Leishmania* metalloprotease GP63 as the mediator of Nramp1 degradation. Conditioned medium from the GP63 knockout strain (LmGP63^−/−^) failed to upregulate hepcidin or degrade Nramp1. Further experiments using conditioned medium from both the wild type and LmGP63^−/−^ strain revealed that GP63 depletes macrophage DICER1, impairing maturation of miR-122, a negative regulator of hepcidin. Consistent with these *in vitro* results, the LmGP63^−/−^ strain, unlike its wild type counterpart, was unable to deplete DICER1, induce hepcidin expression or suppress Nramp1 in infected BALB/c mice. Collectively, we uncover a novel role for *L. major*-secreted GP63 in targeting the host DICER1/miR-122 axis to trigger hepcidin expression and Nramp1 degradation, facilitating iron acquisition by the parasite.

*Leishmania* spp. are protozoan parasites of medical significance belonging to the Trypanosomatidae family. They cause a spectrum of diseases collectively known as leishmaniasis, with symptoms ranging from self-healing cutaneous lesions to potentially fatal visceral infection, known as kala-azar (1). With an estimated 0.7 to 1 million new cases occurring annually, leishmaniasis continues to be a significant global health burden (1, 2). Lack of a vaccine and increasing resistance to the few existing drugs highlight the need for better understanding of the disease pathogenesis, which could lead to the development of alternative therapies (3, 4).

During its dimorphic lifecycle, *Leishmania* alternates between a sand fly vector and its mammalian host. In the midgut of the sand fly, the parasite proliferates in its promastigote form before being transmitted to the host during a blood meal. Following transmission, *Leishmania* promastigotes are rapidly engulfed by host macrophages where they initially remain entrapped within a phagosome, which gradually matures into acidic phagolysosome. Exposure to the phagolysosomal environment drives transformation of long, flagellated, and highly motile promastigotes into rounded amastigotes with short, nonmotile flagella (5, 6). Apart from being acidic, this free radical-rich phagolysosomal compartment has limited nutrient availability, posing a significant challenge to the survival of intracellular *Leishmania* (7, 8). How the parasite hijacks host cell machinery to metabolically adapt to such adverse conditions remains to be fully understood. In this context, investigating *Leishmania’*s iron acquisition strategy within the phagolysosomal niche is of utmost importance.

Iron is an essential micronutrient for all life forms, including *Leishmania*. It acts as a cofactor in several key metalloenzymes and is therefore crucial for the proliferation of intracellular parasites (9, 10). Thus, it is not surprising that *Leishmania amazonensis* lacking the ferrous iron transporter LIT1 failed to replicate within macrophages and lost its ability to cause infection in mice (11). However, despite its indispensability, free iron is highly toxic due to its participation in the Fenton reaction, which generates reactive hydroxyl radicals (12). Therefore, iron availability is tightly regulated in mammalian cells and intracellular *Leishmania* must overcome macrophage’s nutritional immunity to access host iron pool (13, 14). Macrophage iron content is controlled by the interplay of ferritin and ferroportin. Ferritin stores excess iron in a nontoxic, bioavailable form in the cytosol, while ferroportin, located on the plasma membrane exports iron out of the cell to maintain cellular iron homeostasis (15, 16). Iron uptake in macrophages occurs through two main pathways: transferrin-receptor-mediated endocytosis and phagocytosis of senescent RBCs, both delivering iron to the lysosome/phagolysosome for recycling (17, 18). Of particular interest is the natural resistance-associated macrophage protein 1 (Nramp1, also known as SLC11A1), an iron exporter located on the endo/lysosomal membrane that effluxes iron from the phagolysosomes, limiting its availability in this compartment (19, 20, 21).

The *Nramp1* gene was originally identified as the *Lsh* locus through positional cloning as a key determinant of resistance against unrelated intracellular pathogens like *Mycobacteria*, *Salmonella* and *Leishmania* (19, 22). A naturally occurring G169D point mutation in the transmembrane domain of Nramp1 disrupts its proper maturation, rendering inbred mouse strains carrying this mutation more susceptible to these infections (19, 23). While Nramp1 is primarily expressed in phagocytic cells of the myeloid lineage, particularly macrophages, it has also been detected in neurons and microglial cells of the mouse brain (24, 25). Nramp1 co-localizes with late endosomal/lysosomal markers in macrophages and is also recruited to the maturing phagosome (20, 26, 27). Studies in *Dictyostelium* and mammalian macrophage cell have established the role of Nramp1 in mediating efflux of ferrous iron from the phagosome (21, 28, 29). This facilitates efficient recycling of hemoglobin-derived iron in macrophages (30). In addition to iron, Nramp1 has also been shown to extrude manganese from the phagosomal compartment in a pH-dependent manner (31). Another study demonstrated that Nramp1 restricts *Salmonella* growth by depriving it of magnesium (32). Thus, it has been proposed that Nramp1 confers nutritional immunity by limiting the availability of iron and other divalent metals to invading pathogens (33).

Despite its critical role in determining the outcome of host-pathogen interaction, whether Nramp1 levels are modulated during infection remained unknown until recently. We investigated this in *Leishmania major* infected macrophages and observed that at 12 h post infection, Nramp1 levels were significantly reduced but were restored to normalcy by 30 h (34). Consistent with its role as a phagosomal iron exporter, Nramp1 depletion was associated with increased phagolysosomal iron content and a higher intracellular parasite burden. Further, we showed that this infection-induced depletion of Nramp1 was caused by ubiquitin-proteasomal degradation, mediated by its interaction with the iron-regulatory peptide hormone hepcidin, whose expression was markedly upregulated during infection (34). It was evident from these results that *L. major* targets Nramp1 to inhibit iron efflux from its replication niche, the phagolysosome. However, the key question remained: how does *L. major* infection trigger hepcidin expression and target Nramp1 for proteasomal degradation? Taking cue from an intriguing observation that Nramp1 depletion was observed even in bystander uninfected cells in close proximity to *L. major* infected macrophages, we hypothesized that a parasite-secreted factor might be driving this process. To test this, we treated macrophages with *L. major* conditioned media, which induced proteasomal degradation of Nramp1 to a similar extent as direct infection. This discovery set off a series of experiments that culminated in the identification of GP63 as the causative secretory factor. Compelling evidence for this came from the observation that conditioned medium from GP63-deficient parasites failed to cause Nramp1 degradation. GP63 is a zinc-dependent metalloprotease of *Leishmania* that facilitates immune evasion by targeting multiple host proteins; however, until now it has not been reported to target any iron transporter in macrophage (35, 36). Our *in vitro* and *in vivo* animal infection experiments collectively reveal that by depleting DICER1/miR-122, GP63 drives hepcidin upregulation in macrophages, leading to proteasomal degradation of Nramp1. This unique strategy enables *L. major* to subvert host iron homeostasis for its own benefit.

## Results

### Treatment of macrophage cells with *L. major* conditioned media results in proteasomal degradation of Nramp1

**Figure 1:**
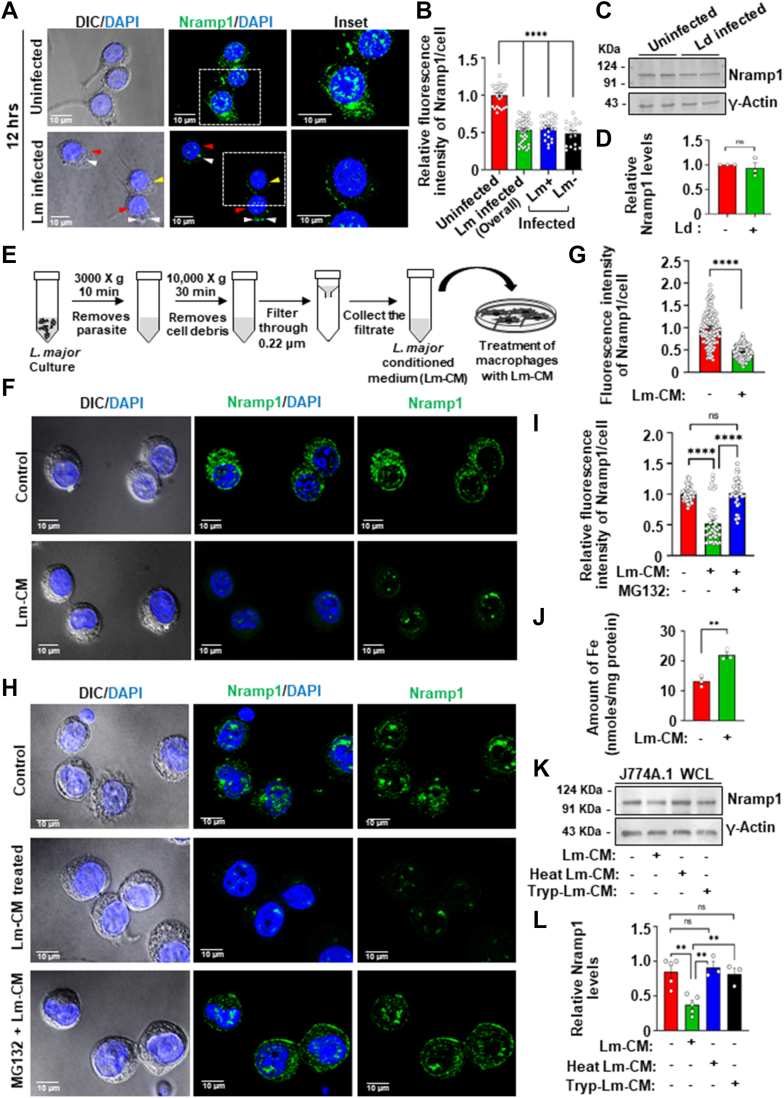
**Treatment with *Leishmania major* conditioned media depleted Nramp1 and elevated endo/lysosomal iron content in macrophages.***A*, Nramp1 was visualized by immunostaining with anti-Nramp1 (*green*) in uninfected or *L. major* (Lm)-infected J774A.1 macrophages. Nuclei were stained with DAPI (*blue*). DIC/DAPI panel shows the presence of intracellular parasites (smaller nuclei, indicated by *white arrows*) in infected cells (marked by *red arrows*). *Yellow arrows* mark the uninfected cells. The insets are zoomed regions marked by *boxes*. Images were acquired with Leica SP8 confocal, 63× objective. *B*, quantification of the Nramp1 fluorescence intensities from uninfected and Lm-infected (overall) macrophages is shown in bar diagram. For the Lm-infected macrophages, fluorescence intensities were separately analyzed for bystander uninfected cells (Lm-) and the infected cells (Lm+). The data are expressed as means ± SEM (Uninfected, n = 25; *L. major* infected (overall), n = 30; Lm+, n = 25; Lm-, n = 18 cells from N = 3 independent experiments). *C*, representative western blots of Nramp1 and γ-actin (loading control) in whole cell lysates (WCL) from either uninfected or *Leishmania donovani* infected J774A.1 macrophages. *D*, bar diagram quantifying Nramp1 band intensities normalized to γ-actin. Data are presented as mean ± SEM from at least three independent experiments. *E*, schematic illustration showing the preparation of *L. major* conditioned medium (Lm-CM). *F*, Nramp1 was visualized by immunostaining with anti-Nramp1 (*green*) in J774A.1 macrophages treated for 12 h with *L. major* conditioned medium (Lm-CM) or with M199 medium only (control). Nuclei were stained with DAPI (*blue*) and the DIC/DAPI panel shows the overall cell morphology. Images were acquired with Carl Zeiss Apotome.2 microscope, 63× objective. *G*, quantification of the Nramp1 fluorescence intensities in the respective images shown in bar diagram. The data are expressed as means ± SEM (at least 150 cells from N = 3 independent experiments were analyzed). *H*, Nramp1 immunostaining (*green*) in J774A.1 macrophages treated for 12 h with just M199 media (control) or with Lm-CM or Lm-CM + 1 μm MG132 (macrophages were pretreated with MG132 prior to Lm-CM treatment). Nuclei were stained with DAPI (*blue*) and the DIC/DAPI panel shows the overall cell morphology. Images were acquired with Carl Zeiss Apotome.2 microscope, 63× objective. *I*, quantification of the Nramp1 fluorescence intensities in the respective images shown in bar diagram. Values expressed as means ± SEM (at least 40 cells from N = 3 independent experiments were analyzed). *J*, quantification of endo/lysosomal iron in J774A.1 macrophages treated for 12 h with just M199 medium (−) or with Lm-CM (+). The data are presented as means ± SEMs from three independent experiments. *K*, representative western blots of Nramp1 and γ-actin (loading control) on J774A.1 macrophage whole cell lysates (WCLs) prepared from either control cells (treated just with M199 media) or cells treated for 12 h with Lm-CM, heat-inactivated Lm-CM or trypsinized Lm-CM. *L*, bar diagram showing the quantification of Nramp1 band densities in the respective samples normalized to γ-actin. Values expressed as means ± SEMs from at least three independent experiments. In all bar diagrams, individual values are shown as *small circles*. n.s., nonsignificant; ∗∗∗∗*p* ≤ 0.0001, ∗∗*p* ≤ 0.01, estimated by two-tailed unpaired Student’s *t* test or one-way ANOVA. DAPI, 4′,6-diamidino-2-phenylindole; DIC, differential interference contrast.

Recently, we reported that *L. major* infection in macrophages caused a significant reduction in Nramp1 protein levels at 12 h post infection. This correlated with a simultaneous increase in phagolysosomal iron content and a higher intracellular parasite burden (34). Consistent with our previous findings, *L. major* infection in J774A.1 macrophages resulted in overall ∼ 2 folds reduction in Nramp1 protein levels, as evidenced by the immunofluorescence results (Fig. 1, *A* and *B*). Interestingly, *Leishmania donovani* infection did not cause any detectable reduction in Nramp1 levels (Fig. 1, *C* and *D*). Such *Leishmania* species-specific modulation of Nramp1 explains prior observations that, although Nramp1, originally identified as the *Lsh* locus, is a proven determinant of resistance against *L. donovani*, it does not restrict *L. major* replication (37, 38). Since our infection protocol routinely yields 65 to 70% infection rate, we were curious to analyze the Nramp1 levels in the bystander uninfected macrophages located in proximity to *L. major* infected cells. Careful image analysis revealed that even the bystander uninfected cells (Lm-) exhibited a similar reduction in Nramp1 levels as their neighboring infected macrophages (Lm+) (Fig. 1*A* inset and B; and Fig. S1*A*). These intriguing data suggested that a secretory factor of the parasite could be involved in reducing the Nramp1 levels in macrophage cells. To investigate whether a secretory factor of *Leishmania* can indeed cause reduction of Nramp1 levels in host macrophages, we cultured *L. major* promastigotes, collected the conditioned media (Lm-CM) containing the parasite’s secretory factors and treated macrophage cells with it (Fig. 1*E*). By microscopic observation and western blot analysis using antibodies against known cytosolic proteins of *Leishmania*, we confirmed that the collected Lm-CM was devoid of any intact parasite or their cytosolic content (Fig. S1, *B* and *C*). Treatment of J774A.1 macrophages with Lm-CM for 12 h led to ∼ 2 folds reduction in the Nramp1 levels, as demonstrated by immunofluorescence microscopy (Fig. 1, *F* and *G*). This intriguing finding was validated in primary peritoneal macrophages derived from BALB/c mice, which also showed a similar reduction in the Nramp1 levels upon Lm-CM treatment (Fig. S1, *D* and *E*). Quantitative RT-PCR (qRT-PCR) data demonstrated that Lm-CM-mediated reduction in Nramp1 levels was not due to transcriptional downregulation of Nramp1 (Fig. S1*F*). However**,** as revealed by our immunofluorescence and western blot data, depletion of Nramp1 could be prevented by treating macrophage cells with the proteasome inhibitor MG132, suggesting involvement of the proteasomal degradation pathway in the process (Figs. 1, *H*, *I* and S1, *G*, *H*). In agreement with our previous finding with *L. major* infection, we observed ∼ 1.7-folds increase in the endo/lysosomal iron content in the Lm-CM-treated J774A.1 macrophages compared to the untreated control (Fig. 1*J*). Interestingly, from our western blot data it was evident that Lm-CM lost its ability to cause Nramp1 degradation when it was preheated at 95 °C or preincubated with trypsin (Fig. 1, *K* and *L*). These results suggest that a heat and protease-sensitive secreted factor (most likely a protein) of the parasite is responsible for Nramp1 degradation in host macrophages.

### Treatment of *L*. *major* conditioned media with metalloprotease inhibitors abrogated its ability to cause Nramp1 degradation

**Figure 2:**
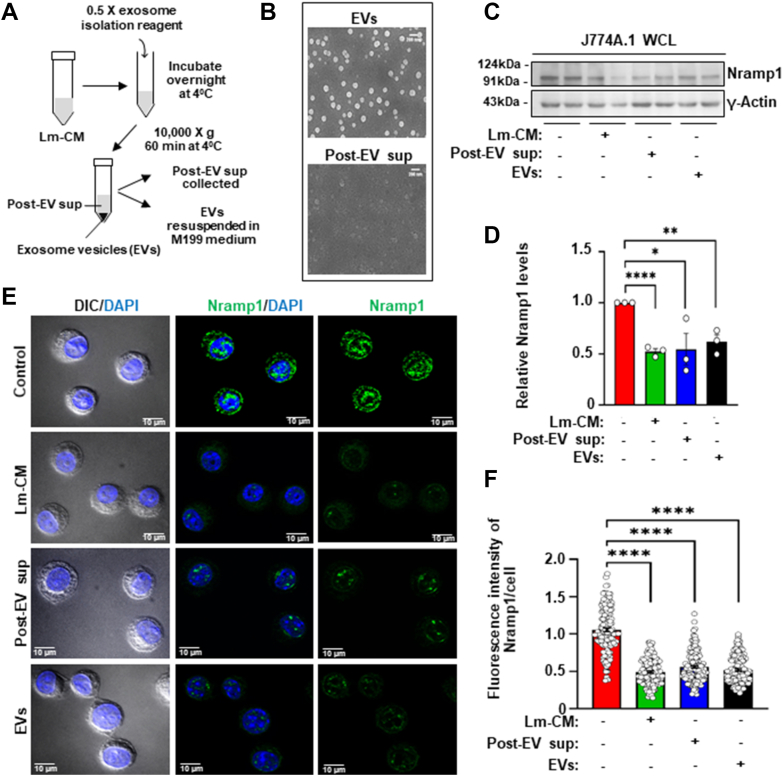
**Treatment with exosomal and nonexosomal fractions of *Leishmania major* conditioned media depleted Nramp1 levels in macrophages.***A*, schematic illustration of isolation of extracellular vesicles (EVs) from Lm-CM. *B*, representative SEM images of EV fraction (*upper* image) and post-EV supernatant fraction (*lower* image) isolated from Lm-CM. The scale bars represent 200 nm. *C*, representative western blots of Nramp1 and γ-actin (loading control) on J774A.1 macrophage whole cell lysates (WCL) obtained from control macrophages (treated just with M199 media) or macrophages incubated for 12 h with Lm-CM, post-EV supernatant or EVs fraction. *D*, bar diagram showing the quantification of Nramp1 band densities in the respective samples normalized to γ-actin. Values expressed as means ± SEMs from three independent experiments. *E*, Nramp1 immunostaining (*green*) in J774A.1 macrophages treated for 12 h with just M199 media (control) or Lm-CM, post-EV supernatant or EVs. Nuclei were stained with DAPI (*blue*) and the DIC/DAPI panel shows the overall cell morphology. Images were acquired with Carl Zeiss Apotome.2 microscope, 63× objective. *F*, quantification of the Nramp1 fluorescence intensities in the respective images shown in bar diagram. Values are expressed as means ± SEMs (at least 147 cells from N = 3 independent experiments were analyzed). In all bar diagrams, individual values are shown as *small circles*. ∗∗∗∗*p* ≤ 0.0001, ∗∗*p* ≤ 0.01, ∗*p* ≤ 0.05, estimated by two-tailed unpaired Student’s *t* test. DAPI, 4′,6-diamidino-2-phenylindole; DIC, differential interference contrast; Lm-CM*, Leishmania major* conditioned medium

**Figure 3:**
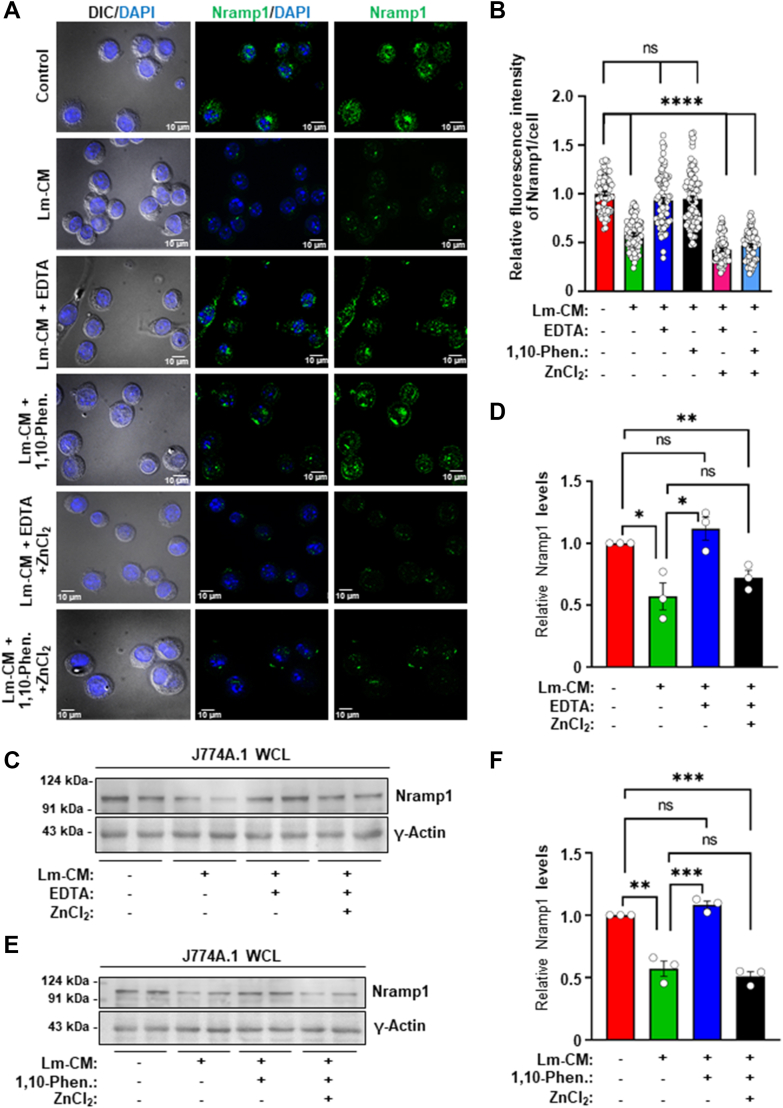
**Metalloprotease inhibitors abrogated the ability of *Leishmania major* conditioned media to cause Nramp1 depletion.***A*, Nramp1 immunostaining (*green*) in J774A.1 macrophages treated for 12 h with just M199 media (control) or Lm-CM, Lm-CM + 1 mM EDTA, Lm-CM + 1 mM 1,10-Phenanthroline, Lm-CM + 1 mM EDTA + 1 mM ZnCl_2_, Lm-CM + 1 mM 1,10- phenanthroline + 1 mM ZnCl_2_. Nuclei were stained with DAPI (*blue*) and the DIC/DAPI panel shows the overall cell morphology. Images were acquired with Carl Zeiss Apotome.2 microscope, 63× objective. *B*, quantification of the Nramp1 fluorescence intensities in the respective images shown in bar diagram. Values are expressed as means ± SEMs (at least 75 cells from N = 3 independent experiments were analyzed). *C*, representative western blots of Nramp1 and γ-actin (loading control) on J774A.1 macrophage whole cell lysates (WCL) obtained from untreated macrophages (treated just with M199 media) or macrophages treated for 12 h with Lm-CM or Lm-CM + 1 mM EDTA or Lm-CM + 1 mM EDTA + 1 mM ZnCl_2_. *D*, bar diagram showing the quantification of Nramp1 band densities in the respective samples normalized to γ-actin. Values expressed as means ± SEMs from N = 3 independent experiments. *E*, representative western blots of Nramp1 and γ-actin (loading control) on J774A.1 macrophage whole cell lysates (WCL) obtained from untreated macrophages (treated just with M199 media) or macrophages treated for 12 h with Lm-CM or Lm-CM + 1 mM 1,10-phenanthroline or Lm-CM + 1 mM 1,10-phenanthroline + 1 mM ZnCl_2_. *F*, bar diagram showing the quantification of Nramp1 band densities in the respective samples normalized to γ-actin. Values expressed as means ± SEMs from N = 3 independent experiments. In all bar diagrams, individual values are shown as *small circles*. n.s., nonsignificant; ∗∗∗∗*p* ≤ 0.0001,∗∗∗*p* ≤ 0.001,∗∗*p* ≤ 0.01, ∗*p* ≤ 0.05 estimated by two-tailed unpaired Student’s *t* test or one-way ANOVA. DAPI, 4′,6-diamidino-2-phenylindole; DIC, differential interference contrast; Lm-CM*, Leishmania major* conditioned medium.

*Leishmania* secretes a wide variety of proteins either in free form, or packed within exosomal vesicles (EVs) (39, 40). So, we were curious to know whether this unknown secretory protein of *L. major* responsible for Nramp1 degradation is released in its free form or bound to exosomes. Using an exosome isolation kit, we isolated the EVs from the Lm-CM and suspended them in M199 media. Alongside, the post-EV supernatant was also collected (Fig. 2*A*). Authenticity of both the fractions was confirmed by scanning electron microscope (SEM) (Fig. 2*B*). Interestingly, our western blot and immunofluorescence data demonstrated that treatment of J774A.1 macrophages with either EVs or the post-EV supernatant fraction resulted in a significant reduction in Nramp1 levels, similar to the effects observed with Lm-CM treatment (Fig. 2, *C*–*F*). Exoproteome analysis of *Leishmania infantum* revealed that only a limited number of secreted proteins are present in both exosomal and nonexosomal fractions, with GP63, a zinc-dependent metalloprotease, being the most abundant among them (39). Taking cue from this, we analyzed the potential role of GP63 in Nramp1 degradation by pharmacological inhibition approach. For this, we preincubated the Lm-CM with the zinc-chelating metalloprotease inhibitors EDTA or 1,10-phenanthroline and then treated the macrophage cells with it (41). Our immunofluorescence as well as western blot data showed that preincubation with EDTA or 1,10-phenanthroline abrogated Lm-CM’s ability to cause Nramp1 degradation. However, exogenous addition of zinc restored this ability in EDTA/1,10-phenanthroline-treated Lm-CM (Fig. 3, *A*–*F*). It may be noted that treatment of macrophage cells with EDTA, 1,10-phenanthroline or ZnCl_2_ alone did not cause any change in the Nramp1 levels (Fig. S2, *A* and *B*). These results suggest that the zinc-chelator-sensitive GP63 present in Lm-CM may be responsible for Nramp1 degradation in macrophages.

### Conditioned media from the GP63^−/−^*L. major* failed to cause Nramp1 degradation

**Figure 4:**
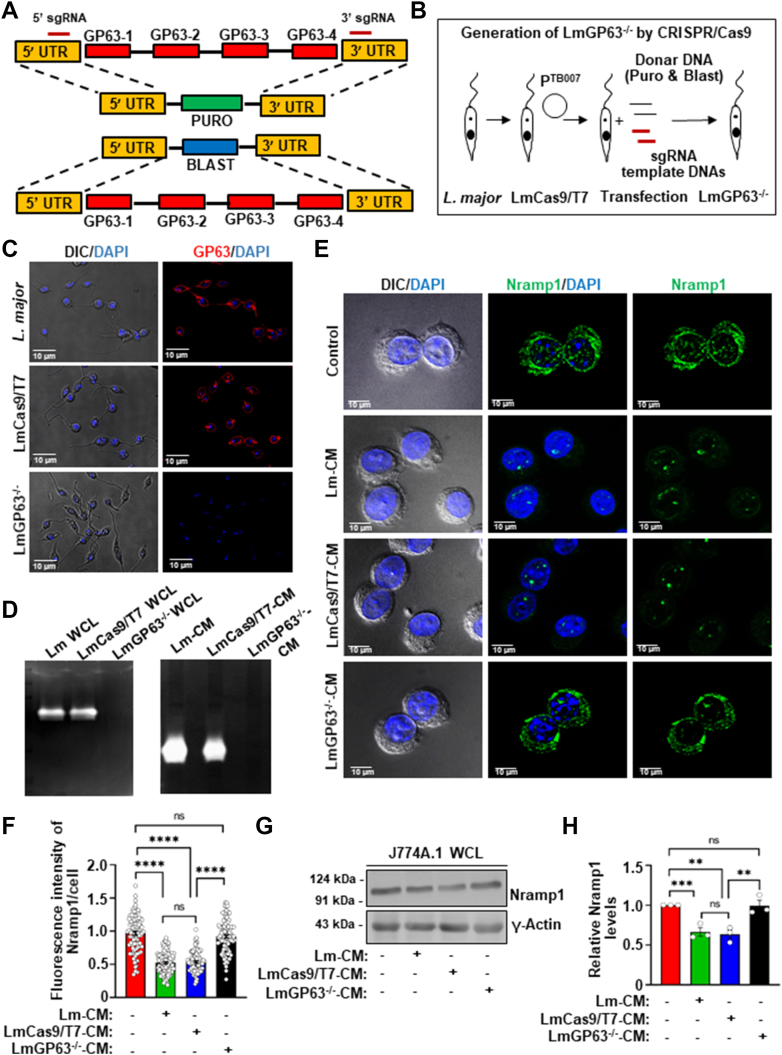
**Conditioned media from the GP63 knockout *Leishmania major* (LmGP63^−/−^) failed to deplete Nramp1 in macrophages.***A* and *B*, schematic illustrations of the GP63 locus in the *L. major* genome and the strategy adopted to generate LmGP63^−/−^ strain using CRISPR/Cas9 technique. *C*, GP63 immunostaining (*red*) with anti-GP63 in wild type *L. major*, LmCas9/T7, and LmGP63^−/−^ promastigotes. Nuclei were stained with DAPI (*blue*) and the DIC/DAPI panel shows the overall cell morphology. Images were acquired with Leica SP8 confocal, 63× objective. *D*, representative gelatin zymograms showing the absence of GP63 activity (absence of *white* bands, indicating gelatin proteolysis) in LmGP63^−/−^ WCL compared to *L. major* WCL and LmCas9/T7 WCL (left zymogram) and in conditioned media from the LmGP63^−/−^ strain (LmGP63^−/−^CM) compared to Lm-CM and LmCas9/T7-CM (right zymogram). *E*, Nramp1 was visualized by immunostaining with anti-Nramp1 (*green*) in J774A.1 macrophages treated for 12 h with M199 media only (control) or with Lm-CM, LmCas9/T7-CM or LmGP63^−/−^CM. Nuclei were stained with DAPI (*blue*) and the DIC/DAPI panel shows the overall cell morphology. Images were acquired with Carl Zeiss Apotome.2 microscope, 63× objective. *F*, quantification of the Nramp1 fluorescence intensities in the respective images shown in bar diagram. Values are expressed as means ± SEMs (at least 90 cells from N = 3 independent experiments were analyzed). *G*, representative western blots of Nramp1 and γ-actin (loading control) on J774A.1 macrophage whole cell lysates (WCL) obtained from untreated macrophages (treated just with M199 media) or macrophages treated for 12 h with Lm-CM, LmCas9/T7-CM or LmGP63^−/−^CM. *H*, bar diagram showing the quantification of Nramp1 band densities in the respective samples normalized to γ-actin. Values are expressed as means ± SEM from three independent experiments. In all bar diagrams, individual values are shown as *small circles*. n.s., nonsignificant; ∗∗∗∗*p* ≤ 0.0001, ∗∗∗*p* ≤ 0.001,∗∗*p* ≤ 0.01 estimated by two-tailed unpaired Student’s *t* test. DAPI, 4′,6-diamidino-2-phenylindole; DIC, differential interference contrast; Lm-CM*, Leishmania major* conditioned medium.

To obtain definitive evidence that GP63 is indeed responsible for degradation of Nramp1, we planned to generate a GP63 knockout *L. major* strain (LmGP63^−/−^) using CRISPR-Cas9. According to the *L. major* genome data base, four copies of the GP63 gene are arranged in tandem on chromosome 10 (42). On the background of an engineered *L. major* strain stably expressing Cas9 and T7 RNA polymerase (LmCas9/T7), we aimed to knock out both the alleles of all four copies of GP63 using sgRNAs that specifically targeted the 5′ UTR of GP63-1 and 3′ UTR of GP63 to 4 (Figs. S3*A* and 4, *A*, *B*). Puromycin and blasticidin repair cassettes replaced the cleaved target sequences, resulting in generation of the LmGP63^−/−^ strain, which was selected in the presence of puromycin dihydrochloride and blasticidin S hydrochloride. The authenticity of the LmGP63^−/−^ strain was verified by genomic PCR using appropriate primers sets confirming the presence of puromycin (600 bp product with primers P1/P2) and blasticidin (390 bp product with primers P3/P4) cassettes and absence of the GP63 gene (checked with primers P5/P6) (Fig. S3, *B* and *C*). Complete absence of the GP63 protein in the LmGP63^−/−^ strain was confirmed by immunofluorescence (Fig. 4*C*), flowcytometry (Fig. S3*D*), and gelatin zymography assay (Fig. 4*D*). Macrophages infected with the LmGP63^−/−^ strain showed significantly reduced infectivity and lower intracellular parasite burden compared to those infected with wild-type *L. major* or the LmCas9/T7 strain, consistent with previous reports and in line with GP63’s established role as a virulence factor (Fig. S3, *E* and *F*) (43). Having extensively validated the LmGP63^−/−^ strain, we next collected the CM from it (LmGP63^−/−^CM) and also from two other control strains, wild type *L. major* (Lm-CM) and LmCas9/T7 (LmCas9/T7-CM). Macrophage cells were treated with these three CM in parallel to compare their abilities to cause Nramp1 degradation. Our immunofluorescence (Fig. 4, *E* and *F*) and western blot (Fig. 4, *G* and *H*) data showed that while Lm-CM and LmCas9/T7-CM could induce Nramp1 degradation in macrophages, LmGP63^−/−^CM completely lacked this ability. These results provided a clear evidence that GP63 in Lm-CM is responsible for inducing proteasomal degradation of Nramp1 in host macrophages and prompted us to investigate the underlying mechanism.

### Conditioned media from the GP63^−/−^*L. major* lost the ability to induce hepcidin expression or trigger ubiquitination of Nramp1 in host macrophages

**Figure 5:**
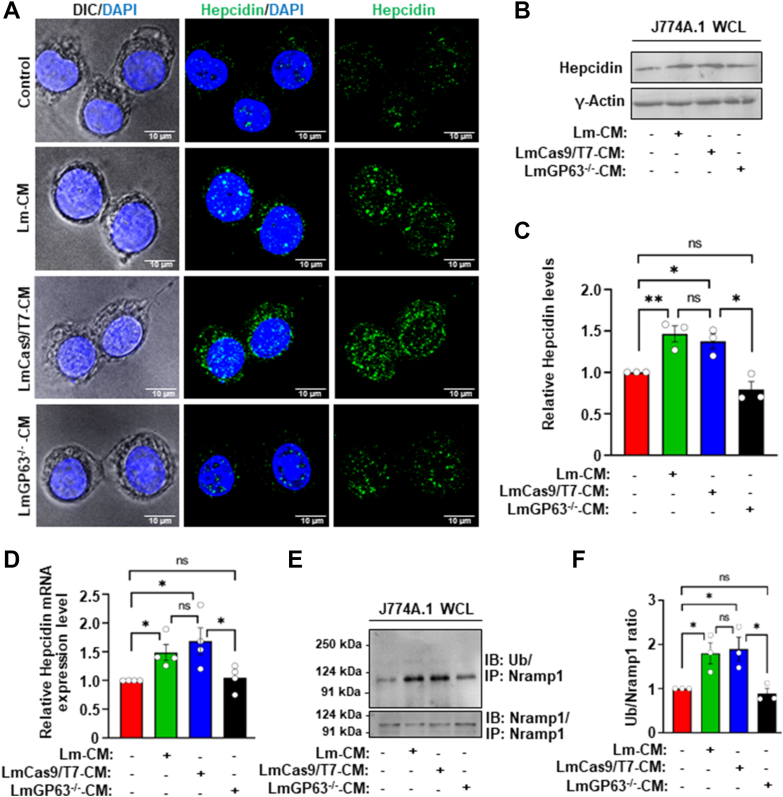
**Conditioned media from the LmGP63^−/−^ strain unable to induce hepcidin expression or trigger Nramp1 ubiquitination in macrophages.***A*, hepcidin was visualized by immunostaining with anti-hepcidin (*green*) in J774A.1 macrophages treated for 12 h with M199 media only (control) or with Lm-CM, LmCas9/T7-CM or LmGP63^−/−^CM. Nuclei were stained with DAPI (*blue*) and the DIC/DAPI panel shows the overall cell morphology. Images were acquired with Carl Zeiss Apotome.2 microscope, 63× objective. *B*, representative western blots of hepcidin and γ-actin (loading control) on J774A.1 macrophage whole cell lysates (WCLs) obtained from untreated macrophages (treated just with M199 media) or macrophages treated for 12 h with Lm-CM, LmCas9/T7-CM or LmGP63^−/−^CM. *C*, bar diagram showing the quantification of hepcidin band densities in the respective samples normalized to γ-actin. Values are expressed as means ± SEMs from three independent experiments. *D*, bar diagram showing qRT-PCR data of relative hepcidin expression in J774A.1 macrophages treated for 12 h with M199 media only (control) or with Lm-CM, LmCas9/T7-CM or LmGP63^−/−^CM. All the measurements were performed using the control cell as reference sample (expression level set to 1.0) and β-actin as an endogenous control gene for normalization. Values are expressed as means ± SEMs from N = 4 independent experiments. *E*, immunoprecipitation (IP) of Nramp1 from J774A.1 macrophage whole cell lysates (WCL) obtained from untreated macrophages (treated just with M199 media) or macrophages treated for 12 h with Lm-CM, LmCas9/T7-CM or LmGP63^−/−^CM, followed by probing with anti-ubiquitin antibody (*top* panel) or with anti-Nramp1 antibody (*bottom* panel). *F*, bar diagram showing the quantification of ubiquitin band densities in the respective samples normalized to Nramp1. Values are expressed as means ± SEMs from three independent experiments. In all bar diagrams, individual values are shown as *small circles*. n.s., nonsignificant; ∗∗*p* ≤ 0.01, ∗*p* ≤ 0.05, estimated by two-tailed unpaired Student’s *t* test. DAPI, 4′,6-diamidino-2-phenylindole; DIC, differential interference contrast; Lm-CM*, Leishmania major* conditioned medium.

Previously, we reported that infection of macrophage cells with *L. major* led to transcriptional upregulation of hepcidin, which in-turn promoted ubiquitination and proteasomal degradation of Nramp1 (34). Since CM from the GP63^−/−^ *L. major* was unable to cause proteasomal degradation of Nramp1, we sought to determine whether this is due to its inability to induce hepcidin expression or trigger ubiquitination of Nramp1. Consistent with our previous findings in *L. major*-infected macrophages, we found that macrophage cells treated with Lm-CM or LmCas9/T7-CM exhibited significant upregulation of hepcidin at both protein (Fig. 5, *A*–*C*) and mRNA levels (Fig. 5*D*), along with increased Nramp1 ubiquitination (Fig. 5, *E* and *F*). Interestingly, treatment with LmGP63^−/−^CM neither led to hepcidin upregulation nor caused Nramp1 ubiquitination (Fig. 5, *A*–*F*). This suggests that GP63 in Lm-CM plays a pivotal role in promoting hepcidin expression, ultimately driving Nramp1 ubiquitination and its proteasomal degradation, thereby enriching iron content in the endo/lysosomal compartment. To assess the physiological relevance of hepcidin levels in determining the outcome of *L. major* infection, we examined whether blocking its expression in macrophages alters intracellular parasite survival. For this, we transcriptionally inhibited hepcidin with heparin prior to infecting macrophages with *L. major*. This treatment reduced intracellular parasite load by nearly twofold at 12, 24, and 36 h post infection (p.i.) compared to untreated controls, indicating that suppression of hepcidin limits parasite survival and/or replication within host macrophages (Fig. S4*A*). Interestingly, heparin treatment also caused a slight (∼1.2 fold) reduction in infectivity (Fig. S4*B*). Together, these results establish the functional role of hepcidin in regulating *L. major* infection and prompted us to investigate the mechanism by which GP63 controls its expression.

### GP63 in *L. major* conditioned media targets DICER1 to inhibit maturation of miR-122, a negative regulator of hepcidin

**Figure 6:**
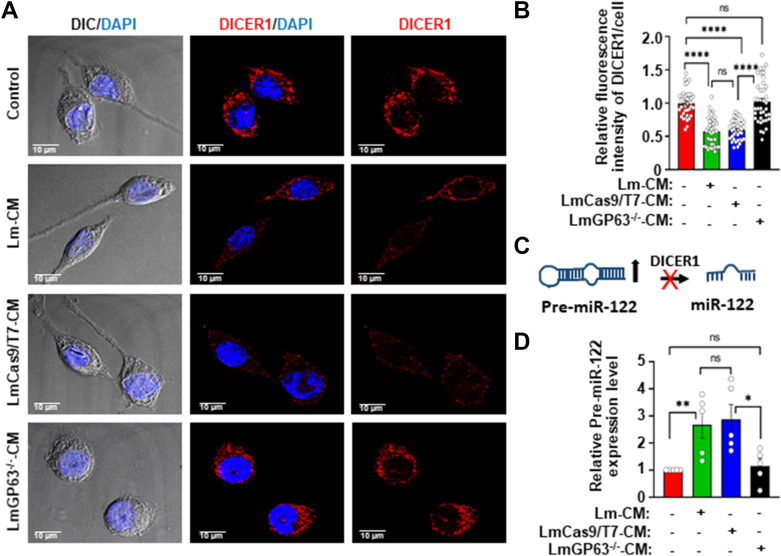
***Leishmania major* GP63 targets DICER1 to inhibit miR-122 maturation in macrophages.***A*, DICER1 was visualized by immunostaining with anti-DICER1 (*red*) in J774A.1 macrophages treated for 12 h with M199 media only (control) or with Lm-CM, LmCas9/T7-CM, or LmGP63^−/−^CM. Nuclei were stained with DAPI (*blue*) and the DIC/DAPI panel shows the overall cell morphology. Images were acquired with Leica SP8 confocal, 63× objective. *B*, quantification of the DICER1 fluorescence intensities in the respective images shown in bar diagram. Values are expressed as means ± SEMs (at least 39 cells from N = 3 independent experiments were analyzed). *C*, schematic representation of DICER1-mediated processing of pre-miR-122 to mature miR-122. As shown in the figure, depletion of DICER1 is expected to increase the abundance of pre-miR-122. *D*, bar diagram showing qRT-PCR data of relative pre-miR-122 mRNA levels in J774A.1 macrophages treated for 12 h with M199 media only (control) or with Lm-CM, LmCas9/T7-CM or LmGP63^−/−^CM. All the measurements were performed using the control cell as reference sample (expression level set to 1.0) and β-actin as an endogenous control gene for normalization. Values are expressed as means ± SEMs from N = 5 independent experiments. In all bar diagrams, individual values are shown as *small circles*. n.s., nonsignificant; ∗∗∗∗*p* ≤ 0.0001, ∗∗*p* ≤ 0.01, ∗*p* ≤ 0.05 estimated by two-tailed unpaired Student’s *t* test. DAPI, 4′,6-diamidino-2-phenylindole; DIC, differential interference contrast; qRT-PCR, quantitative RT-PCR

Hepcidin expression in the liver has been shown to be negatively regulated by miR-122 (44). In an unrelated study, it was observed that purified GP63 from *L. donovani* extract cleaves DICER1, an endoribonuclease essential for processing of precursor microRNAs (pre-miRNAs) into its mature and functional form (45). These prior studies prompted us to check whether treating macrophage cells with Lm-CM could deplete DICER1 levels and, consequently, inhibit the processing of pre-miR-122 in a GP63-dependent manner. As shown in Figure 6, *A* and *B*, we found a significant depletion of DICER1 in macrophage cells treated with Lm-CM or LmCas9/T7-CM, but not in those cells treated with LmGP63^−/−^CM, suggesting that GP63 promotes the loss of DICER1. To determine whether this observation also holds true during infection, we next examined DICER1 levels in *L. major*-infected macrophages at different time points post-infection (6–72 h p,i.). Consistent with the results with Lm-CM, infected macrophages also showed loss of DICER1, which was most pronounced at 12 h p.i., a time point that coincided with the time point for maximal Nramp1 depletion (Fig. S5, *A*–*D*). A previous study reported that GP63 levels are much lower at both the mRNA and protein levels in *L. major* amastigotes compared to promastigotes (46). In agreement, our data also revealed that GP63 expression was abundant at 12 h p.i. but declined sharply by 72 h, supporting the conclusion that GP63 primarily exerts its effect on DICER1 during the early phase of infection (Fig. S5*E*). Since depletion of DICER1 is expected to inhibit the process of microRNA maturation, we checked whether treatment of macrophages with Lm-CM or LmCas9/T7-CM leads to an accumulation of pre-miR-122 due to the processing defect (Fig. 6*C*). Our qRT-PCR data confirmed that treatment of macrophages with Lm-CM or LmCas9/T7-CM resulted in a significant accumulation of pre-miR-122, indicating a block in miR-122 maturation. However, this impairment was not observed in macrophages treated with LmGP63^−/−^CM, where pre-miR-122 levels remained unchanged (Fig. 6*D*). Collectively, these results indicate that GP63-mediated depletion of DICER1 is responsible for the inhibition of miR-122 maturation. Given that miR-122 is a negative regulator of hepcidin expression, we propose that GP63 present in Lm-CM induces hepcidin expression in host macrophages by targeting the DICER1/miR-122 axis, thereby facilitating proteasomal degradation of Nramp1.

### GP63 acts through the DICER1/hepcidin axis to lower Nramp1 levels in *L. major -*infected BALB/c mice

**Figure 7:**
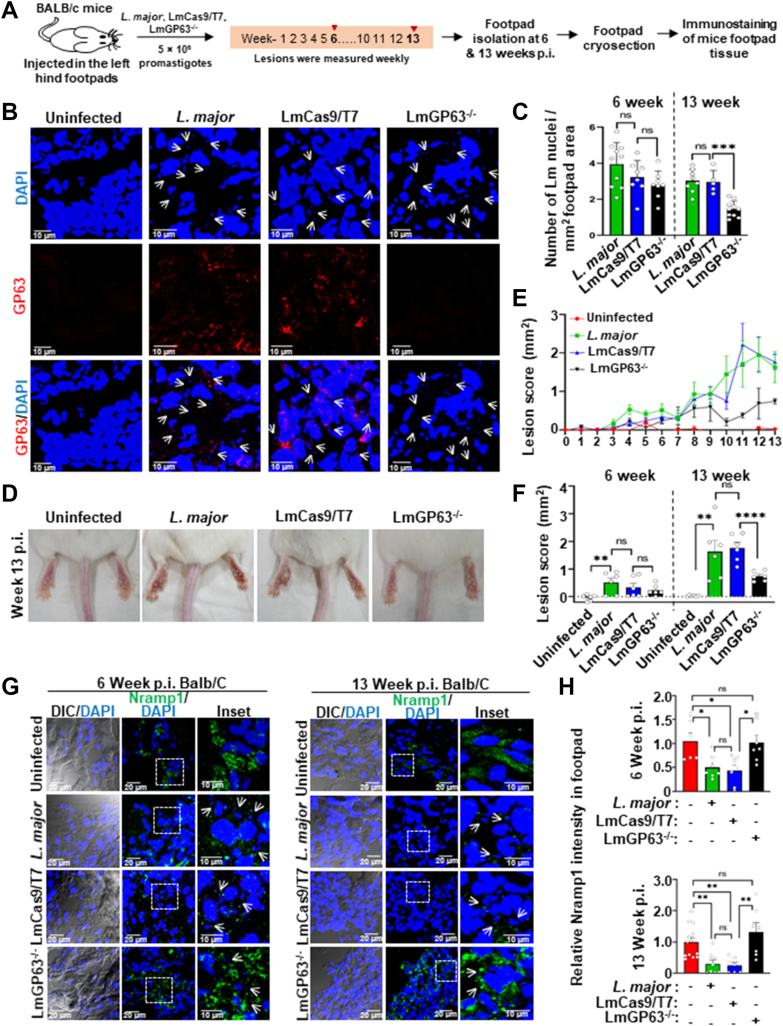
**Infection of BALB/c mice with LmGP63^−/−^ parasite failed to suppress Nramp1.***A*, schematic illustration of the infection protocol of BALB/c mice with wild type *Leishmania major*, LmCas9/T7, or LmGP63^−/−^ strains. *B*, immunofluorescence staining for GP63 (*red*) in the footpad cryosections of BALB/c mouse that were either uninfected or infected subcutaneously in the left hind footpad with 5 × 106 stationary-phase promastigotes of wild type *L. major*, LmCas9/T7 or LmGP63*−/−* strains at 6 weeks post infection (p.i.). Nuclei were stained with DAPI (*blue*) and small *Leishmania* nuclei are marked with *white arrows*. *C*, bar diagram showing the number of DAPI-stained parasite nuclei per mm2 area of footpad tissue of BALB/c mice that were infected with wild type *L. major*, LmCas9/T7, or LmGP63^−/−^ strains at 6 or 13 weeks p.i. Quantification was performed from maximum intensity projections (MIP) of confocal images, considering an average nuclear area of ∼2.5 μm^2^. Individual values are represented as *small circles*. *D*, representative images of the footpad of BALB/c mice that were either uninfected or infected with wild type *L. major*, LmCas9/T7, or LmGP63^−/−^ strains at 13 weeks p.i. *E*, footpad swelling was monitored weekly by measuring the width and thickness, and the lesion scores were plotted through 13 weeks p.i. *F*, bar diagram showing footpad lesion scores of BALB/c mice that were either uninfected or infected with wild type *L. major*, LmCas9/T7, or LmGP63^−/−^ strains at 6 and 13 weeks p.i. Data are expressed as mean ± SEM (n = 6 mice per group). *G*, immunofluorescence staining for Nramp1 (*green*) in the footpad cryosections of BALB/c mouse that were either uninfected or infected with wild type *L. major*, LmCas9/T7 or LmGP63^−/−^ strains. Tissues were harvested at 6 (*left panel*) or 13 (*right panel*) weeks p.i. Nuclei were stained with DAPI (*blue*) and small *Leishmania* nuclei are marked with *white arrows*. *H*, quantification of the Nramp1 fluorescence intensities in the respective mice footpad cryosections. *Toppanel* shown the data for 6 weeks p.i. while the 13 weeks p.i. data are in the *bottom panel*. Images were acquired with Leica SP8 confocal, 63× objective. Values are expressed as means ± SEMs (N = 5 mice). In all bar diagrams, individual values are shown as *small circles*. n.s., nonsignificant; ∗∗∗*p* ≤ 0.001, ∗∗*p* ≤ 0.01, ∗*p* ≤ 0.05, estimated by two-tailed unpaired Student’s *t* test. DAPI, 4′,6-diamidino-2-phenylindole.

**Figure 8:**
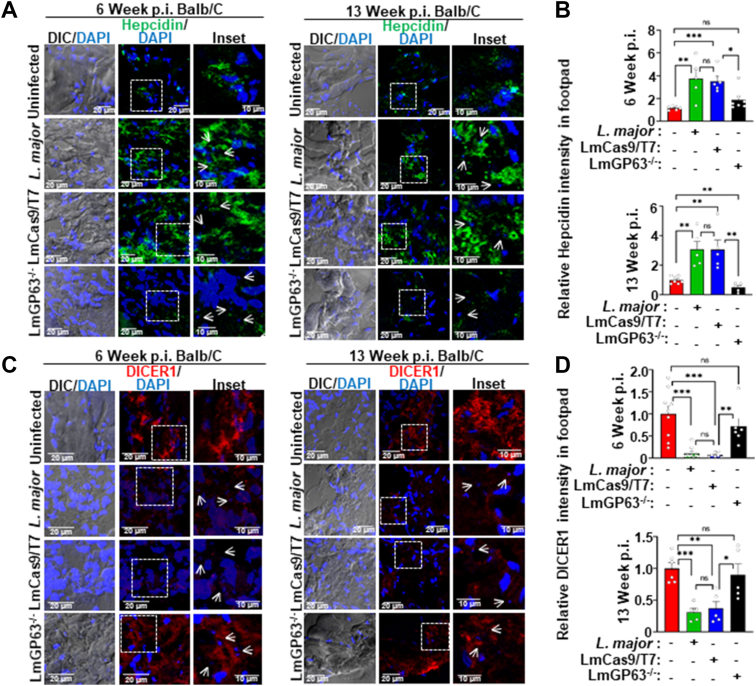
**LmGP63^−/−^ infection in BALB/c mice was unable to induce hepcidin expression or deplete DICER1.***A*, immunofluorescence staining for hepcidin (*green*) in the footpad cryosections of BALB/c mouse that were either uninfected or infected with wild type *Leishmania major*, LmCas9/T7 or LmGP63^−/−^ strains. Tissues were harvested at 6 (*left panel*) or 13 (*right panel*) weeks p.i. Nuclei were stained with DAPI (*blue*) and small *Leishmania* nuclei are marked with *white arrows*. *B*, quantification of the hepcidin fluorescence intensities in the respective mice footpad cryosections. *Top panel* shown the data for 6 weeks p.i. while the 13 weeks p.i. data are in the *bottom panel*. Values are expressed as means ± SEMs (N = 5 mice). *C*, immunofluorescence staining for DICER1 (*red*) in the footpad cryosections of BALB/c mouse that were either uninfected or infected with wild type *L. major*, LmCas9/T7 or LmGP63^−/−^ strains. Tissues were harvested at 6 (*left panel*) or 13 (*right panel*) weeks p.i. Nuclei were stained with DAPI (*blue*) and small Leishmania nuclei are marked with *white arrows*. *D*, quantification of the DICER1 fluorescence intensities in the respective mice footpad cryosections. *Top panel* shows the data for 6 weeks p.i. while the 13 weeks p.i. data are in the *bottom panel*. Images were acquired with Leica SP8 confocal, 63× objective. Values are expressed as means ± SEMs (N = 5 mice). In all bar diagrams, individual values are shown as *small circles*. n.s., nonsignificant; ∗∗∗*p* ≤ 0.001, ∗∗*p* ≤ 0.01, ∗*p* ≤ 0.05, estimated by two-tailed unpaired Student’s *t* test. DAPI, 4′,6-diamidino-2-phenylindole; p.i., post infection.

We next asked whether the mechanism by which GP63 in Lm-CM regulates Nramp1 levels in cultured macrophage cells is also operational in an *in vivo* infection model. As illustrated in Figure 7*A*, BALB/c mice were infected in the footpad with wild-type *L. major* (LmWT), LmCas9/T7, or LmGP63^−/−^ strains, and immunofluorescence analysis of Nramp1, DICER1, and hepcidin was performed on cryosectioned footpads at 6 and 13 weeks p.i. First, we confirmed the presence of GP63 in footpad sections of the mice infected with LmWT or LmCas9/T7 strains and as expected, no GP63 signal was detected in mice infected with the LmGP63^−/−^ strain (Fig. 7*B*). At 6 weeks p.i., mice infected with the LmGP63^−/−^ strain showed parasite loads and lesion scores almost comparable to LmWT and LmCas9/T7. By 13 weeks p.i., however, LmGP63^−/−^ infection resulted in ∼2-fold lower parasite burden and significantly reduced lesion development (Fig. 7, *C*–*F*). These results are consistent with a previous report demonstrating that compared to wild type *L. major* infection, GP63-deficient parasites exhibited a mild delay in lesion formation during early infection (∼5 weeks), which became more pronounced over time (47). Staining of footpad sections with Nramp1 antibody revealed a significant reduction in Nramp1 levels in mice infected with LmWT or LmCas9/T7 strains at 6 and 13 weeks p.i. compared to the uninfected control, whereas the LmGP63^−/−^ strain failed to deplete Nramp1 (Fig. 7, *G* and *H*). Colocalization of Nramp1 with CD32 (FcR), a macrophage-specific marker, confirmed that Nramp1 is exclusively expressed in the macrophages of the mouse footpad (Fig. S6*A*). Consistent with our previous report, footpad iron levels were significantly elevated in LmWT- and LmCas9/T7-infected mice at 13 weeks p.i. compared with uninfected controls (48). In contrast, LmGP63^−/−^ infection led to only a modest but statistically nonsignificant increase in footpad iron, markedly lower than that observed in LmWT and LmCas9/T7 infections (Fig. S6*B*). These data unambiguously establishes the role of GP63 in augmenting the iron level at the site of the infection. Next, we checked for the DICER1 and hepcidin levels in cryosectioned footpad sections of uninfected BALB/c mice or those infected with LmWT, LmCas9/T7, or LmGP63^−/−^ strains. In agreement with our *in vitro* data, we observed a significant reduction in the DICER1 levels with a concomitant surge of hepcidin levels in the footpad sections of the mice infected with LmWT or LmCas9/T7 strains at 6- and 13-weeks post infection compared to uninfected mice. Footpad sections of the LmGP63^−/−^ mice showed neither a depletion of DICER1 levels nor an increase in hepcidin levels, and these levels were comparable to those in uninfected mice (Fig. 8, *A*–*D*). Collectively, these findings provide an unambiguous *in vivo* validation that GP63 indeed acts through the DICER1/hepcidin axis to lower Nramp1 levels in host macrophages.

## Discussion

**Figure 9:**
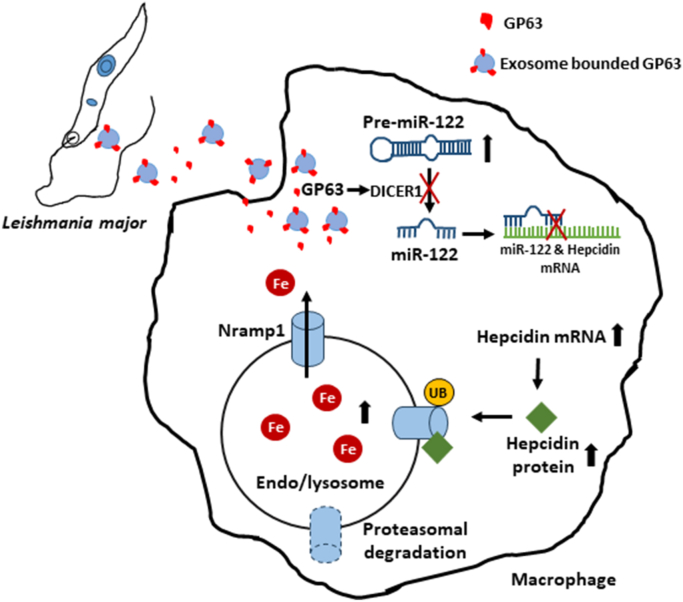
**Schematic representation of GP63 mediated Nramp1 degradation mechanism.***Leishmania major*-secreted GP63 targets DICER1 in host macrophages, blocking maturation of miRNA-122, a known negative regulator of hepcidin. This triggers hepcidin expression, ultimately leading to hepcidin-mediated proteasomal degradation of Nramp1 and enhanced endo/lysosomal iron content.

Nutritional immunity is a vital host defense system that deprives pathogens of essential micronutrients, such as iron, to hinder their growth and limit infection (49, 50). A key player in this process is Nramp1, which actively efflux iron from macrophage phagolysosomes, reducing its availability to engulfed pathogens. However, *L. major* has evolved a sophisticated strategy to counteract this by causing hepcidin-mediated proteasomal degradation of Nramp1 upon infection. Here, we uncover a crucial role of the parasite-secreted factor GP63 in driving this process. We demonstrate that GP63 of *L. major* targets the DICER1/miR-122 axis in host macrophages to trigger hepcidin expression, ultimately leading to depletion of Nramp1 and enhanced phagolysosomal iron content (Fig. 9).

Our quest to identify the parasite-secreted factor responsible for causing Nramp1 depletion was prompted by an interesting observation that Nramp1 levels were diminished not just in *L. major* infected cells but also in neighboring uninfected ones. Such unanticipated bystander effect suggested that the parasite’s influence on Nramp1 extended beyond direct infection, possibly through a diffusible factor capable of modulating the host environment. This notion was further fueled by the fact that a large number of secreted proteins constitute the *Leishmania* exoproteome, and that treatment of macrophages with *Leishmania mexicana*-derived exoproteome has been shown to modulate signaling pathways, including inhibition of nitric oxide production (51). Consistent with our hypothesis, we found that treating macrophages with Lm-CM could cause proteasomal degradation of Nramp1. However, identifying the secretory factor responsible for Nramp1 depletion was a challenge. Multiple studies have shown that the primary mode of protein secretion in *Leishmania* occurs *via* EVs, and only a minor fraction of the secreted proteins contains an N-terminal signal peptide for secretion in free form (40, 52). Interestingly, analysis of the *L. infantum* exoproteome revealed a distinct subset of proteins present in both the exosomal fraction and as free forms, with GP63, a zinc-dependent metalloprotease, being the most abundant among them (39). This prior observation, combined with our data showing that both exosomal and non-exosomal fractions of the Lm-CM retained Nramp1-depleting activity, suggested that GP63 could be the elusive secretory factor driving this effect. We provided definitive evidence for this by exposing macrophages to Lm-CM pretreated with GP63 inhibitors or to CM from the GP63^−/−^ *L. major*, both of which failed to induce Nramp1 degradation.

Having confirmed that GP63 in Lm-CM is indeed responsible for proteasomal degradation of Nramp1, the next daunting task was to delineate the underlying mechanism. GP63 was originally identified as a major surface glycoprotein of *Leishmania* with Zn-dependent proteolytic activity. It was proposed to play roles in parasite’s attachment to macrophages while also protecting it from phagolysosomal degradation (41, 53). Infection with GP63-deficient parasites resulted in a delayed onset of lesion formation in mice, establishing its role as a virulence factor (47). Subsequent studies confirmed that GP63 is also released into the extracellular space through autoproteolysis at the cell surface as well as *via* exosomal secretion (54). Interestingly, GP63 was also detected within exosomes released from the *L. mexicana* infected macrophages (55). Exosomal GP63 is rapidly internalized by recipient macrophages *via* a lipid raft-dependent mechanism and play vital roles in host-pathogen communication (56). Utilizing its proteolytic activity, GP63 is known to alter macrophage signaling and the innate immune response (36). Prior studies have identified protein tyrosine phosphatases (PTPs) as targets of GP63, leading to their activation and subsequent modulation of *MAPK and JAK-STAT* signaling pathways in macrophages (56). Membrane fusion regulators such as Synaptotagmin XI, VAMP8, and Syntaxin-5 were also reported as host substrates for GP63 (35, 57, 58). Purified GP63 from *L. donovani* was shown to cleave the microRNA processing enzyme DICER1, heterologously expressed in HEK293T cells. This DICER1-targeting ability enables *L. donovani* to supress miR-122 in mouse liver, resulting in reduced serum cholesterol levels through downregulation of miR-122-regulated cholesterol biosynthesis genes (45). A more recent study demonstrated that *L. donovani* GP63 cleaves poly(rC)-binding proteins 1 and 2 (PCB1, and PCB2), which act as chaperones for iron loading into iron storage protein ferritin (59). However, GP63 has not yet been shown to directly or indirectly target any iron transport protein of the host cell.

Since Lm-CM-induced depletion of Nramp1 was found to be mediated by proteasomal degradation, the possibility of Nramp1 being a direct target of GP63 was ruled out. This is also supported by our previous finding that *L. major* infection reduces Nramp1 levels through the ubiquitin-proteasome pathway. Earlier, we also demonstrated that *L. major* infection in macrophages led to upregulation of the iron-regulatory peptide hormone hepcidin, which physically interacts with Nramp1. Inhibition of hepcidin transcription prevented Nramp1 degradation, suggesting that Nramp1 depletion in *L. major* infected macrophages is hepcidin-dependent (34). Interestingly, we observed that while treatment of macrophages with Lm-CM significantly enhanced hepcidin expression and Nramp1 ubiquitination, CM from the GP63^−/−^ *L. major* lacked these properties. Together, these findings suggest that GP63 in Lm-CM indirectly promotes Nramp1 depletion by enhancing hepcidin expression.

Hepcidin, primarily produced by the hepatocytes, regulates iron release into the plasma by binding to ferroportin, the iron exporter located on the plasma membrane of target cells, leading to its internalization and degradation (60). In response to excess iron in circulation, hepatic hepcidin is transcriptionally activated *via* the BMP6-SMAD pathway. On the hepatocyte surface, bone morphogenetic protein 6 (BMP6) interacts with BMP receptors and the coreceptor hemojuvelin (HJV) to activate SMAD signaling. Phosphorylated SMAD1/5/8 forms a heteromeric complex with SMAD4, which translocates to the nucleus, binds to the *HAMP* (hepcidin) gene promoter, and induces its transcription (61, 62, 63). In addition, hepcidin expression is positively regulated by the hereditary hemochromatosis protein (HFE), which signals through transferrin receptor 2 (TfR2) or BMP type 1 receptor ALK3 (64). Interestingly, the microRNA miR-122 has been found to negatively regulate hepcidin production in hepatocytes by inhibiting transcription of *Hjv* and *Hfe*, key activators of hepcidin expression (44). These previous reports on hepcidin regulation in hepatic cells prompted us to ask whether similar regulatory circuit exists in macrophages and if *L. major* GP63 can tweak it to stimulate hepcidin expression. Since, microRNA-processing enzyme DICER1 has been identified as a substrate for *L. donovani* GP63, we were curious to examine the status of DICER1 and miR-122 in macrophage cells treated with Lm-CM or CM from the GP63^−/−^ *L. major* (45). Strikingly, treatment with Lm-CM, but not with CM from the GP63^−/−^ parasite, caused significant depletion of DICER1 and impaired processing of pre-miR-122 to mature miR-122 in macrophages. Given that miR-122 is a known repressor of hepcidin, the GP63-mediated processing defect of miR-122 provides a mechanistic explanation for the upregulation of hepcidin in Lm-CM-treated macrophages and the consequent hepcidin-mediated proteasomal degradation of Nramp1 (44). Does the mechanism by which GP63 in Lm-CM targets DICER1/miR-122/hepcidin axis to deplete Nramp1 levels in cultured macrophage cells also operates under actual infection conditions? This was tested in an animal model of *L. major* infection in BALB/c mice. Compared to wild type *L. major*, the GP63 deficient parasite (LmGP63^−/−^) clearly lacked the ability to deplete DICER1, induce hepcidin expression or reduce Nramp1 levels in the infected mouse footpad, providing *in vivo* validation for our *in vitro* observations.

To summarize, we uncover a previously unrecognized regulatory axis controlling Nramp1 levels in macrophages, which is hijacked by *L. major* GP63 to sequester iron within phagolysosomal compartment, thereby supporting parasite survival. It was interesting to note that Nramp1 depletion peaked at ∼12 h p.i. but began to recover by ∼30 h, suggesting that the parasite primarily targets Nramp1 during the early phase to secure iron and establish infection. This transient suppression likely contributes to the subsequent decline in parasite load in macrophages observed over the next 36 h of infection. In line with these observations, GP63 expression was also high at 12 h p.i. but declined sharply by 72 h, consistent with previous reports showing that GP63 levels are markedly reduced in amastigotes compared to promastigotes (46). Together, these findings indicate that GP63-driven Nramp1 depletion constitutes an early infection strategy, allowing *L. major* to transiently remodel the phagolysosomal environment before host defense regain control. Intriguingly, *L. donovani* infection did not reduce Nramp1 levels, unlike *L. major*, which utilizes a GP63/DICER1/miR-122/hepcidin pathway to deplete Nramp1. This species-specific difference likely reflects distinct iron acquisition strategies. Although both parasites encode GP63, *L. donovani* harbors fewer gene copies, which may underlie its inability to trigger Nramp1 degradation (65). These findings reconcile earlier genetic studies showing that Nramp1 confers resistance to *L. donovani* but does not restrict *L. major* replication (37, 38). It remains to be seen if this miR-122/hepcidin-mediated post translational regulation of Nramp1 has other physiological implications beyond influencing the infection outcome. The role of Nramp1 in facilitating efficient iron recycling during erythrophagocytosis is well-established (30, 66). Notably, overexpression of Nramp1 has been shown to elevate redox-active labile iron pool in the cytosol, likely due to enhanced iron export from the phagolysosomal compartment (30). The labile iron pool being a potent inducer of oxidative stress and cell death (12), it would be intriguing to explore whether miR-122/hepcidin-mediated depletion of Nramp1 serves as a protective mechanism to mitigate iron-induced cytotoxicity, particularly during episodes of heightened erythrophagocytosis.

## Experimental procedures

Reagents, antibodies, and primers/plasmids used in this study are listed in Tables S1, S2 and S3, respectively. Unless otherwise specified, all other reagents were obtained from Sigma-Aldrich.

### *Leishmania* culture and preparation of conditioned medium

*L. major* promastigotes (strain 5ASKH, kindly gifted by Dr Subrata Adak, CSIR-IICB, Kolkata) and *L. donovani* (strain MHOM/IN/1983/AG83, kindly provided by Dr Nahid Ali, CSIR-IICB, Kolkata) were grown at 26 °C in M199 medium supplemented with 23.5 mM Hepes, 10 μg/ml hemin, 150 μg/ml folic acid, 0.2 mM adenine, 120 U/ml penicillin, 60 μg/ml gentamycin, 120 μg/ml streptomycin, 4.1 mM sodium bicarbonate (only used for *L. donovani*), and 15% heat-inactivated fetal bovine serum (67). Cas9 and T7 RNA polymerase expressing *L. major* cells were grown similarly with additional supplementation of 50 μg/ml hygromycin. The GP63^−/−^ *L. major* strain was maintained with additional supplementation of 20 μg/ml puromycin and 5 μg/ml blasticidin.

The CM from *L. major* or *L. donovani* promastigotes (wild type or the engineered strains) were prepared from late log phase culture (3.6 × 10^7^ cells/ml) of parasites. The culture was centrifuged at 1200*g* for 10 min at 26 °C to obtain cell free supernatant, which was further centrifuged at 10,000*g* for 30 min to remove any debris. The CM, containing secretory factors, was sterilized by passing through 0.22 μm filter units. The absence of intact parasites or their cytosolic content in the collected CM was confirmed by microscopic observation and western blot using antibodies against known cytosolic proteins of *Leishmania*. Wherever mentioned, the CM were heated at 95 °C for 5 min and then cooled to room temperature before being used to treat macrophages. For protease digestion, trypsin was added to the CM at a final concentration of 0.25%, mixed thoroughly, and incubated at 37 °C for 10 min. Thereafter, a protease inhibitor cocktail was added to inactivate the trypsin prior to using it for treatment of macrophages. For pharmacological inhibition of GP63, the CM were incubated with divalent metal chelators EDTA (1 mM) or 1, 10-phenanthroline (1 mM) at 37 °C for 2 h.

### Infection of macrophages with *L. major* or *L. Donovani* and treatment with conditioned medium

J774A.1 murine macrophage cell line (American Type Culture Collection, ATCC #TIB-67) were cultured in Dulbecco's modified Eagle medium (DMEM) at 37 °C in a humidified CO_2_ (5%) incubator. The medium was supplemented with 2 mM L-glutamine, 100 U/ml penicillin, 100 μg/ml streptomycin, and 10% heat-inactivated fetal bovine serum, and adjusted to pH 7.4. Following our previously published protocol, the lipopolysaccharide-activated macrophages were infected with *L. major* or *L. donovani* at a ratio of 1:30 (34, 67). After indicted time points post infection (*e.g.* 6 h, 12 h, 30 h, 48 h, and 72 h), cells were washed with PBS, fixed with acetone–methanol (1:1), permeabilized, and processed for immunofluorescence staining. Intracellular parasite burden was estimated as described previously (34, 67). Briefly, macrophages were infected with *L. major* promastigotes for 12 h, after which noninternalized parasites were removed by washing with PBS. For 24 and 36 h infections, cells were incubated for an additional 12 or 24 h, respectively, before fixation. Infected cells were fixed with acetone–methanol (1:1) and stained with antifade medium containing 4′,6-diamidino-2-phenylindole (DAPI). Parasite burden was quantified by analyzing at least 100 cells per sample across three biological replicates. Infectivity was expressed as the percentage of infected macrophages relative to the total number of macrophages counted.

For CM treatment, uninfected macrophages (∼6 h post seeding) was incubated for 12 h with *L. major* or *L. donovani* CM [100 μl or 1 ml CM for 1.2 × 10^5^ (for immunofluorescence studies) or 1.2 × 10^6^ (for Western blot experiments) macrophage cells, respectively].

### Isolation of peritoneal macrophage from BALB/c mice

Thioglycolate-elicited peritoneal macrophages were isolated from 6- to 8-week-old BALB/c mice, as described earlier (68). The mice were maintained at the IISER Kolkata animal facility following CCSEA guidelines and the experiment was conducted as per Institutional Animal Ethics Committee (IAEC) approved protocol After 4 days of intraperitoneal injection with 3% Brewer's thioglycolate medium, mice were euthanized, and peritoneal macrophages were extracted using a 20G needle. The collected macrophages were then cultured and treated with *L. major* CM as described above.

### Isolation of EVs from *L. major* conditioned medium

The exosomal vesicles (Lm-EVs) were isolated from the *L. major* CM using an exosome isolation kit following the protocol suggested by the manufacturer. Briefly, 0.5 ml of the total exosome isolation reagent was added to 1 ml of the CM and mixed well by pipetting. Mixture was then incubated overnight at 4 °C and centrifuged at 10,000*g* for 1 h at 4 °C to pellet the Lm-EVs. The Lm-EVs were resuspended in appropriate volume of M199 media or PBS. Alongside, the post-EV supernatant was also collected. Both the fractions (Lm-EVs and post-EV supernatant) were used for the treating the macrophages or SEM.

### SEM of purified exosomal vesicles

For SEM, purified EVs were isolated from *L. major* CM. The EVs were diluted with filtered PBS, and 10 μl of the sample was deposited on a mica sheet, and then incubated at room temperature until dry. The sample was washed with PBS and fixed with 2.5% glutaraldehyde for 40 min at room temperature. Following fixation, the EVs were washed with PBS and further fixed with OsO_4_ for 5 min at room temperature. The EVs were washed twice with PBS and then with Milli-Q water to remove any residual salts. Finally, the EVs were visualized using a Zeiss Supra 55VP SEM.

### Generation of GP63 knockout *L. major* strain (LmGP63^−/−^) by CRISPR/Cas9

LmGP63^−/−^ strain was generated on the background of an *L. major* strain stably expressing Cas9 and T7 RNA polymerase (LmCas9/T7) that was recently developed by us (69). At first, the LmCas9/T7 strain was verified by genomic DNA PCR with Cas9 FP/Cas9 RP and T7 pol FP/T7 pol RP primer sets for amplification of Cas9, T7 RNA polymerase, respectively. The rRNA45 gene, amplified with rRNA45 FP/rRNA45 RP primer set, was used as control (Fig. S3*A*). We planned to knock out both the alleles of all four copies of GP63 following the method described by Beneke *et al.* and as illustrated in Figure 4, *A* and *B* (70). Briefly, 5′ GP63sgRNA and 3′ GP63sgRNA oligonucleotides were used to generate sgRNA templates. These oligonucleotides contained the T7 promoter, sgRNA sequence for specifically targeting 20-nucleotide long sequences at 354 bp upstream of 5′ UTR of GP63-1 ORF or 512 bp downstream of 3′ UTR of GP63-4 ORF and a complementary sequence for the sgRNA scaffold. To amplify the sgRNA templates, PCR reactions were set up using G00 primer (which provides the sgRNA scaffold) and 5′ GP63sgRNA or 3′ GP63sgRNA oligonucleotides. Simultaneously, the puromycin and blasticidin resistance cassettes containing 30-nt overhangs of 5′ UTR of GP63-1 ORF and 3′ UTR of GP63-4 ORF were PCR-amplified using pTBLAST or pTPURO plasmids as templates and GP63-1 5′UTR and GP63-1 3′UTR primer sets. All four PCR products (5′ GP63sgRNA template, 3′ GP63sgRNA template, puromycin, and blasticidin resistance cassettes) were cotransfected into mid-log phase LmCas9/T7 promastigotes by electroporation. Prior to electroporation, the PCR products were heat-sterilized at 94 °C for 5 min. The LmGP63^−/−^ strain, generated upon integration of the puromycin and blasticidin resistance cassettes into the genome at the sgRNA target sites replacing the GP63 ORFs, was selected in presence of 20 μg/ml puromycin and 5 μg/ml blasticidin. The authenticity of the strain was confirmed by various methods as described in the results section.

### Cell lysis and western blot

J774A.1 macrophages were scrapped from tissue culture plates and collected in microfuge tubes whereas suspension culture of *L. major* was taken directly in the microfuge tubes. The cells were then pelleted at 1200g for 5 min, washed twice with ice-cold PBS and then dissolved in lysis buffer (PBS, 1× EDTA-free protease inhibitor cocktail, and 2 mM PMSF). The cells were lysed by sonication to prepare the whole cell lysates (WCL). Soluble proteins from the *L. major* conditioned medium were precipitated using chilled acetone, resuspended in PBS, and denatured in Laemmli buffer. The WCL (macrophage or *L. major*) or suspension of conditioned medium were resolved by SDS gel electrophoresis and transferred to activated polyvinylidene fluoride membrane. The membrane was blocked in 5% skim milk in 0.05% tris-buffered saline with tween-20 and probed primary antibodies (rabbit anti-Nramp1, 1:1000; rabbit anti-γ-actin, 1:4000; rabbit anti-hepcidin, 1: 500; mouse anti-Dicer1, 1:1000; rabbit anti-Ldactin, 1:5000; rabbit anti-tubulin antibody, 1:4000 and rabbit anti-LmPGAM, 1:2000. Following washing, blots were then probed with appropriate horseradish peroxidase-conjugated secondary antibody (goat anti-rabbit, 1:4000 or rabbit anti-mouse, 1:4000). The blots were developed using SuperSignal West Pico Chemiluminescent Substrate, and bands intensities were quantified using ImageJ software, with γ-actin as the loading control.

### Immunofluorescence microscopy

For immunofluorescence studies, macrophages were seeded in 6-well tissue culture plates containing 22 mm coverslips at a density of 1.2 × 10^5^ for 6 hours. For, *L. major* promastigotes, log phase cells were directly mounted on a poly-L-lysin coated glass coverslip. The cells were fixed with acetone: methanol (1:1) for 10 min at room temperature and permeabilized with 0.1% tritonX −100. After two PBS washes, cells were blocked with 0.2% gelatin for 2 h at room temperature. Cells were washed with PBS and incubated with primary antibodies (rabbit anti-Nramp1, 1:100; mouse anti-GP63, 1:600; rabbit anti-Hepcidin, 1: 100; mouse anti-Dicer1 1:100) for 1 h at room temperature. Following another PBS wash, cells were incubated with secondary antibodies (goat anti-rabbit Alexa Fluor 488, 1:800; goat anti-mouse Alexa Fluor 568, 1:800) for 2 h at room temperature. Cells were again washed with PBS and mounted with an antifade mounting media containing DAPI. Images were acquired with Leica SP8 confocal, Carl Zeiss Apotome.2 or Olympus IX-81 epifluorescence microscope using oil immersion 63× or 40× objectives. Relative fluorescence intensities were measured from Z planes that were merged as a maximum intensity projection where region of interest was selected for individual cells using the microscope's software ZEN Blue.

### Flow cytometry

GP63 surface expression in *L. major* promastigotes was analyzed by flow cytometry as previously described (47). Briefly, *L. major* promastigotes (5 × 10^6^ cells) were harvested and washed twice with ice-cold PBS containing 0.5% bovine serum albumin (BSA). The cells were labeled with mouse anti-GP63 primary antibody (1:600) and incubated at 4 °C for 1 h. After primary antibody incubation, the cells were washed twice with containing 0.5% BSA and incubated with goat anti-mouse Alexa Fluor 568 secondary antibody (1:800) at 4 °C for 1 h. The cells were washed again with 1× PBS containing 0.5% BSA and resuspended in 1 ml 1× PBS. GP63 expression was analyzed using a BD FACSVerse flow cytometer.

### Gelatin zymography assay

The activity of GP63 in *L. major* WCL and conditioned medium was measured by gelatin zymography assay (71). *L. major* promastigotes (1 × 10^7^) were washed twice with ice-cold 1× PBS and resuspended in lysis buffer containing 1× PBS, EDTA-free protease inhibitor cocktail, 2 mM PMSF. The cells were sonicated to prepare the WCL. *L. major* WCL or conditioned medium were mixed with loading dye containing glycerol and bromophenol blue and both samples were resolved on 10% SDA-PAGE gel copolymerized with 0.12% fish gelatin. Following electrophoresis, the resolved gel was incubated in a buffer (pH 7.4) containing 50 mM Tris, 5 mM CaCl_2_, 2.5% Triton X and 1 μM ZnCl_2_ for 1 h at room temperature. After incubation, the gels were rinsed in distilled H_2_O, and GP63 activity was detected by incubating overnight in reaction buffer (pH 7.4) containing 50 mM Tris, 5 mM CaCl_2,_ and 1 μM ZnCl_2_ at 37 °C. The gels were visualized by staining with Coomassie blue and subsequently destained to reveal the zones of proteolytic activity.

### Iron quantification in the endo/lysosomal compartments and mice footpad

Endo/lysosomal vesicles were isolated from the macrophage cells using sucrose density gradient centrifugation as described by us previously (34). Briefly, macrophages were washed with ice-cold 1X PBS and resuspended in lysis buffer containing 1X PBS, EDTA-free protease inhibitor cocktail, and 2 mM PMSF. The cell lysate was prepared by passing the suspension through a 26G needle several times, followed by centrifugation at 3000 rpm for 5 min to remove nuclei and unbroken cells. The resulting supernatant was then centrifuged at 12,000 rpm for 6 min. After separating the supernatant in the sucrose gradient, the endo/lysosome-enriched 4% sucrose fraction was collected. The endo/lysosomal fraction was then incubated with iron-releasing reagent (10 mM HCl and 4.5% KMnO4) for 2 h at 60 °C. For iron estimation in mice footpad, the tissues were first weighed, homogenized in iron-releasing reagent by mechanical disruption, and incubated for 2 h at 60 °C then centrifuged to remove debris. The clarified tissue lysate was then used for iron estimation. The iron content (Fe^2+^) of the endo/lysosomal fraction or mice footpad tissue lysate was quantified using a ferrozine assay (34). After cooling all the samples to room temperature, 30 μl iron detection reagent (6.5 mM neocuproine, 6.5 mM ferrozine, 2.5 M ammonium acetate, and 1 M ascorbic acid) was added. The mixture was incubated for 30 min, and the absorbance was measured at 550 nm using a microplate reader. A standard curve was prepared using a different standard concentration of FeCl_3_ (0–300 μM), and sample iron concentration of samples was calculated from the FeCl_3_ standard curve.

### RNA isolation and qRT-PCR

Total RNA was isolated from J774A.1 macrophages using TRIzol reagent. The isolated RNA was treated with DNase1 to remove any DNA contamination. Using Verso cDNA synthesis kit, cDNA were synthesized from 1 μg of the total RNA. Real-time quantitative PCR was performed on 7500 Real-time PCR systems (Applied Biosystems) using SYBR green fluorophore. Transcript levels of Nramp1, Pre-miR-122, and hepcidin were quantified using respective primers. The transcript levels were normalized with respect to β-actin gene expression. The relative expression levels were calculated using the comparative Ct method (72).

### Infection of BALB/c mice with *L. major*

As described by us previously, infection experiments in BALB/c mice were performed at IISER Kolkata animal facility following IAEC-approved protocol (48). Briefly, 6- to 8-weeks-old female BALB/c mice were subcutaneously injected in the left hind footpad with 5 × 10^6^ late stationary-phase *L. major* promastigotes (five mice/group in each of the experimental set). As uninfected control, the mice were injected with PBS. Lesion development was assessed weekly by measuring the width and thickness of the infected footpad using a digital caliper. As an internal control, the same measurements were taken from the uninfected footpad of each mouse. The lesion score was determined using the following formula: (width of infected footpad—width of noninfected footpad) × (thickness of infected footpad - thickness of noninfected footpad).

### Cryosectioning and immunostaining of mice footpads tissue

At 6-weeks and 13-weeks, post infected mice were euthanized and infected mice footpads were harvested and immediately embedded in optimal cutting temperature compound. The embedded tissues were then kept at −20 °C overnight to ensure complete freezing. Subsequently, 5 μm thick cryosections were cut using Leica CM1950 cryostat and sections were taken on the poly-L-lysine coated glass slides. Sections were air-dried and fixed immediately with 4% paraformaldehyde for 10 min, followed by washing with PBS. Tissue sections were then permeabilized with 0.2% Triton X-100 in PBS for 15 min. After washing with PBS (2 times) sections were blocked with 0.2% gelatin in PBS for 30 min and incubated with primary antibodies (rabbit anti-Nramp1, 1:100; mouse anti-GP63, 1:500; rabbit anti-Hepcidin, 1: 100; mouse anti-Dicer1, 1:100; rat anti-FcƔ, 1:100) for 1 h. Sections were then washed with PBS (3 times) and incubated with secondary antibodies for 1.5 h at room temperature. Tissues were finally mounted using Vectashield mounting media with DAPI and imaged in Leica SP8 confocal microscope.

### Statistical analysis

All statistical analyses were performed using either Student’s t test (for comparisons between two groups) or one-way ANOVA (for comparisons among more than two groups). Data are presented as mean ± SEM from at least three independent experiments. Statistical significance was defined as *p* < 0.05, with levels indicated as follows: ∗*p* ≤ 0.05, ∗∗*p* < 0.01, ∗∗∗*p* < 0.001, ∗∗∗∗*p* < 0.0001.

## Data availability

Further information and requests for resources and reagents should be directed to the lead contact, Prof. Rupak Datta (rupakdatta@iiserkol.ac.in).

## Supporting information

Supplemental Information includes Figures S1-S6 and Tables S1-S3.

## Conflict of interest

The authors declare that they have no conflicts of interest with the contents of this article.
