# Supplementary material for: A secreted Leishmania metalloprotease manipulates host iron regulation by targeting the DICER1–miRNA pathway
Source: J Biol Chem. 2025 Oct 22;301(12):110851. doi: 10.1016/j.jbc.2025.110851 (PMC12666551; doi:10.1016/j.jbc.2025.110851)
Supplement: Supporting Figures and Tables [file mmc1.pdf]

Supporting information

Leishmania-secreted GP63 targets DICER1/miR-122/hepcidin axis in host macrophages to deplete Nramp1

Suman Samanta, Sourav Banerjee[#] and Rupak Datta[*]

Department of Biological Sciences, Indian Institute of Science Education and Research (IISER) Kolkata, Mohanpur, West Bengal, INDIA

[*]To whom correspondence to be addressed.

Rupak Datta

E-mail: rupakdatta@iiserkol.ac.in

Tel: +91 033 6136 0000; Extn: 1214

Figure S1 (Related to Figure 1)

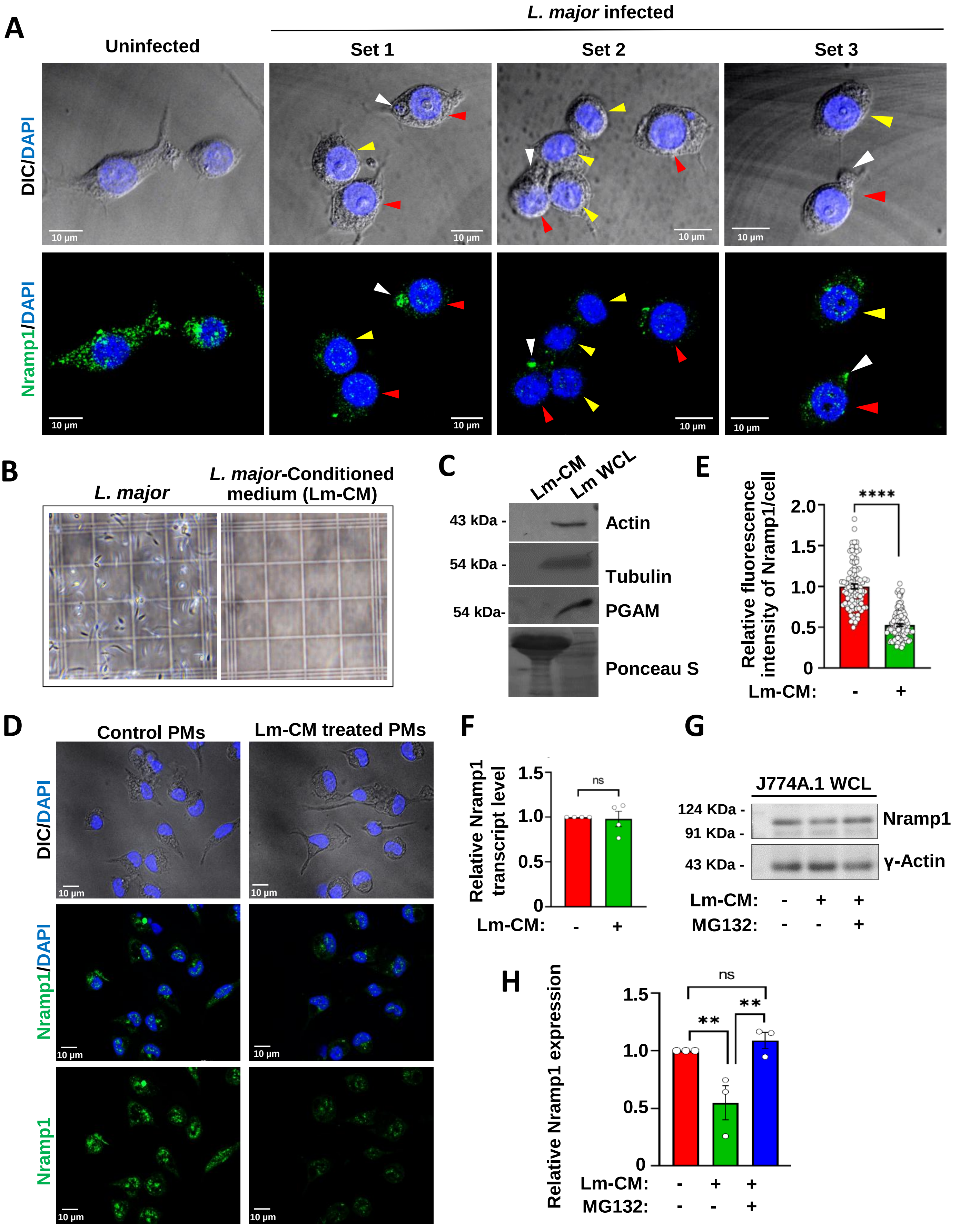

Figure S1. **Infection with *L. major* or treatment with *L. major* conditioned medium induces Nramp1 degradation in peritoneal macrophages (A)** Nramp1 was visualised by immunostaining with anti-Nramp1 (green) in uninfected or *L. major* (Lm)-infected J774A.1 macrophages (3 fields from different experimental sets). Nuclei were stained with DAPI (blue). DIC/DAPI panel shows the presence of intracellular parasites (smaller nuclei, indicated by white arrows) in infected cells In all the three sets of Lm-infected macrophages, decrease in Nramp1 fluorescence intensities were seen in the the infected cells (marked by red arrows) as well as in bystander uninfected cells marked by yellow arrows). Images were acquired with Leica SP8 confocal, 63× objective **(B)** Light microscope images of *L. major* promastigotes and *Leishmania* condition medium (Lm-CM) confirming the absence of any parasite in it. **(C)** Western blot with antibodies against cytosolic proteins of *Leishmania* (actin, tubulin, and PGAM) on Lm-CM or *L. major* whole cell lysates (Lm WCL). Ponceau S stained membrane shows total proteins in Lm-CM and Lm WCL. For Lm WCL, 15mg protein was loaded whereas for Lm-CM ~ 50mg protein was loaded to confirm the absence of any cytosolic proteins of the parasite in Lm-CM. **(D)** Nramp1 was visualised by immunostaining with anti-Nramp1 (green) in BALB/c mice derived peritoneal macrophages treated for 12 hours with *L. major* conditioned medium (Lm-CM) or with M199 medium only (control). Nuclei were stained with DAPI (blue) and the DIC/DAPI panel shows the overall cell morphology. Images were acquired with Carl Zeiss Apotome.2 microscope, 63× objective. **(E)** Quantification of the Nramp1 fluorescence intensities in the respective images shown in bar diagram. The data are expressed as means ± SEM (at least 100 cells from N = 3 independent experiments were analyzed). **(F)** Bar diagram showing qRT-PCR data of relative Nramp1 expression in J774A.1 macrophages treated for 12 hours with M199 media only (-) or with Lm-CM (+). The measurements were performed using the untreated cell as reference sample (expression level set to 1.0) and β-actin as an endogenous control gene for normalization. Values are expressed as means ± SEMs from N = 4 independent experiments. **(G)** Representative western blots of Nramp1 and Ɣ-actin (loading control) on J774A.1 macrophage whole cell lysates (WCL) prepared from either cells treated with just with M199 media (-) or with Lm-CM or Lm-CM + 1µm MG132 (macrophages were pre-treated with MG132 prior to Lm-CM treatment). **(H)** Bar diagram showing the quantification of Nramp1 band densities in the respective samples normalized to Ɣ-actin. Values expressed as means ± SEMs from at least three independent experiments. In all bar diagrams, individual values are shown as small circles. n.s., non-significant; ****$P \leq 0.0001$,**$P \leq 0.01$estimated by two-tailed unpaired Student’s t-test.

Figure S2 (Related to Figure 3)

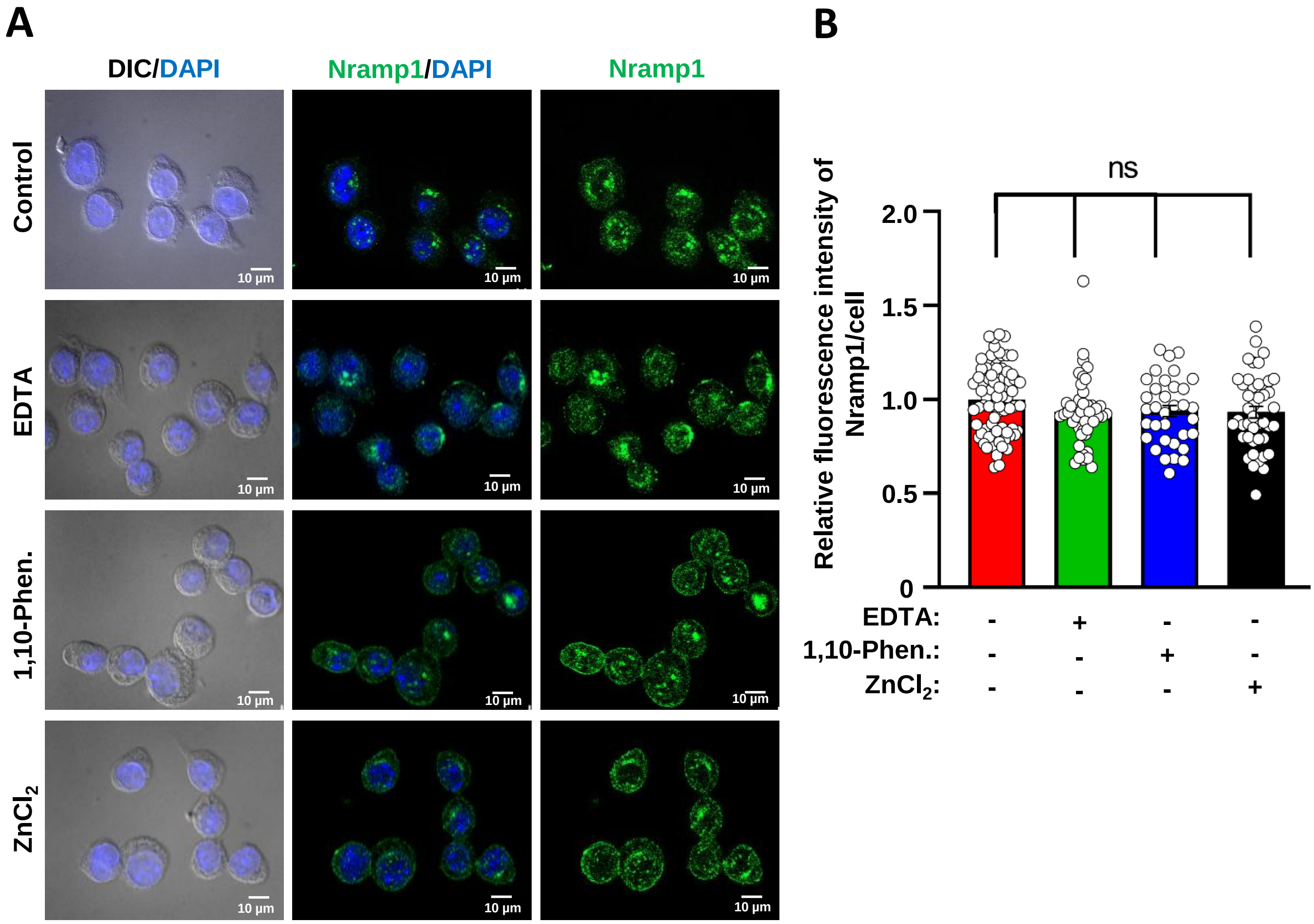

Figure S2. **Effect of EDTA, 1,10-Phenanthroline and $ZnCl_2$ on Nramp1 expression. (A)** Nramp1 immunostaining (green) in J774A.1 macrophages treated for 12 hours with just M199 media (control) or M199 + 1mM EDTA, M199 + 1mM 1,10-Phenanthroline or M199 + 1mM $ZnCl_2$. Nuclei were stained with DAPI (blue) and the DIC/DAPI panel shows the overall cell morphology. Images were acquired with Carl Zeiss Apotome.2 microscope, 63× objective. **(D)** Quantification of the Nramp1 fluorescence intensities in the respective images shown in bar diagram. Values are expressed as means ± SEMs (at least 35 cells from N = 3 independent experiments were analyzed). In the bar diagram, individual values are shown as small circles. n.s., non-significant, estimated by one-way ANOVA.

Figure S3 (Related to Figure 4)

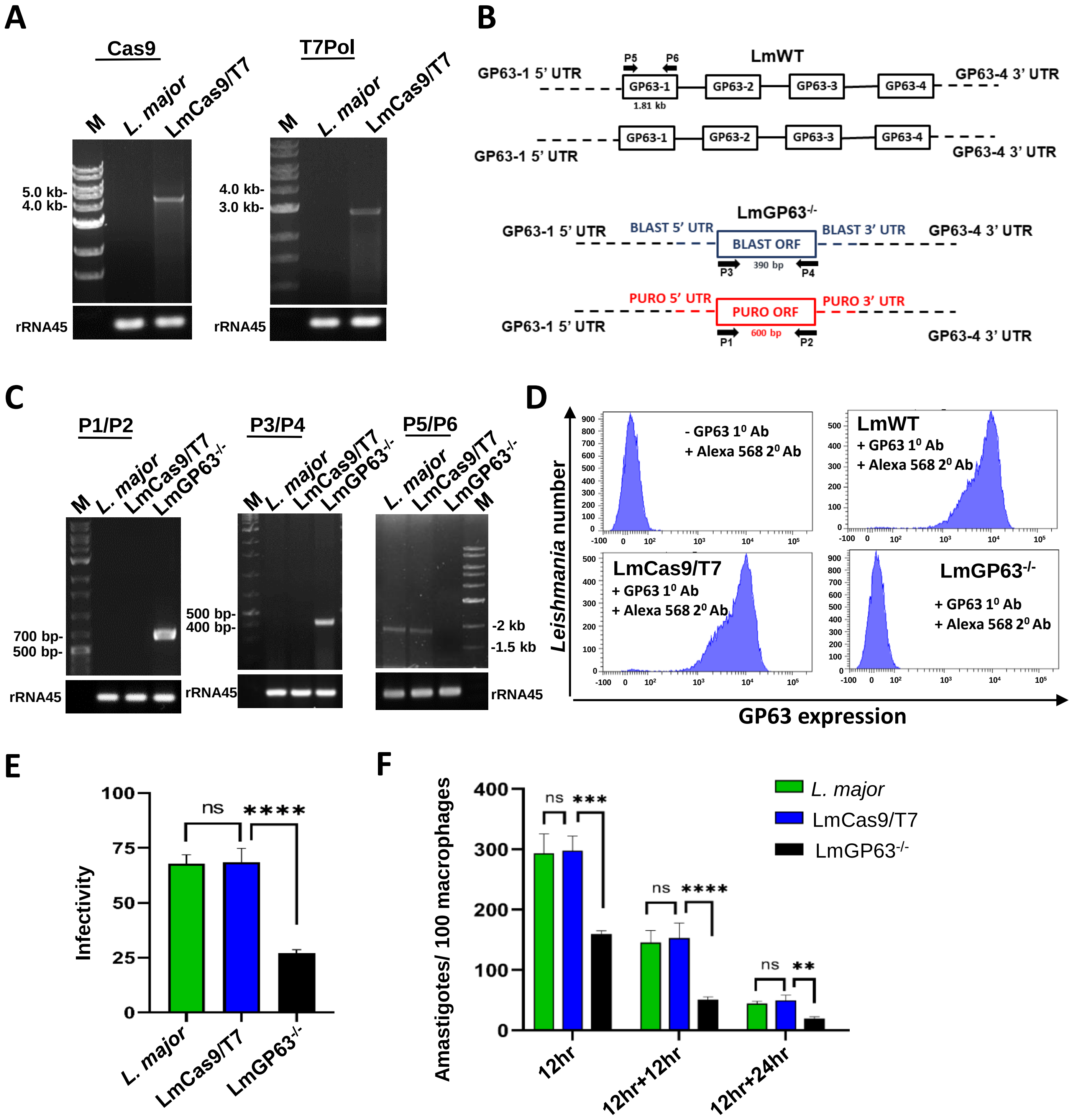

Figure S3. **Verification of the LmCas9/T7 and LmGP63$^{-/-}$ strains. (A)** Agarose gel images showing PCR amplification of the Cas9 and T7 polymerase genes (using gene-specific primers) using LmCas9/T7 genomic DNA as template but not the wild type *L. major* genomic DNA template. **(B)** Schematic representation of the organization of GP63 genes in *L. major* genome. Primers used for the verification of the LmGP63$^{-/-}$ strain are shown. **(C)** Agarose gel images showing genomic DNA PCR results confirming the presence of puromycin (600 bp product with primers P1/P2) and blasticidin (390 bp product with primers P3/P4) cassettes and absence of the GP63 gene (checked with primers P5/P6) in the LmGP63$^{-/-}$ strain. **(D)** FACS analysis of with only Alexa-568-conjugated anti-mouse antibody or mouse anti-GP63 + Alexa-568-conjugated anti-mouse antibody confirming the expression of GP63 in wild type *L. major* and the LmCas9/T7 strain but not in the LmGP63$^{-/-}$ strain. **(E)** Bar diagram showing the infectivity of wild type *L. major*, LmCas9/T7, or LmGP63$^{-/-}$ strains in J774A.1 macrophages at 12 hours post-infection. Infectivity was calculated as the percentage of infected macrophages. At least 100 macrophages were counted per condition. Data represent mean ± SEM from at least three independent experiments.

(F) Bar diagram showing the intracellular parasite burden of wild type *L. major*, LmCas9/T7, or $LmGP63^{-/-}$ strains in macrophages at 12 hours, (12+12) hours and (12+24) hours post-infection. Parasite load, expressed as amastigotes per 100 macrophages, was quantified by counting at least 100 macrophages per condition. Data represent mean ± SEM from three or more independent experiments. n.s., non-significant; ****$P \leq 0.0001$, ***$P \leq 0.001$, **$P \leq 0.01$ estimated by two-tailed unpaired Student's t-test.

Figure S4 (Related to Figure 5)

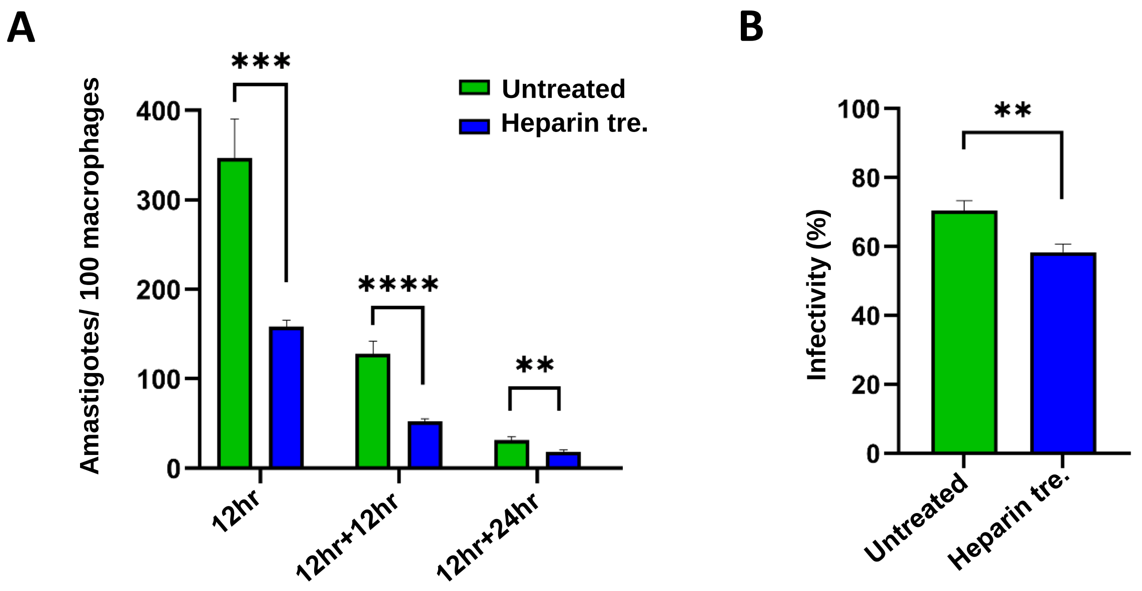

Figure S4. **Heparin treatment reduces *L. major* parasite burden in macrophages. (A)** Bar diagram showing the intracellular parasite burden of *L. major* infected (untreated) and 4 µg/ml heparin-treated *L. major*-infected macrophages at 12 hours, (12+12) hours and (12+24) hours post-infection. Parasite load, expressed as amastigotes per 100 macrophages, was quantified by counting at least 100 macrophages per condition. Data represent mean ± SEM from at least three independent experiments. **(B)** Bar diagram showing the infectivity of *L. major* infected (untreated) and 4 µg/ml heparin-treated *L. major*-infected macrophage at 12 hours post-infection. Infectivity was calculated as the percentage of infected macrophages after incubation with promastigotes for 12 hours. At least 100 macrophages were counted per condition. Data represent mean ± SEM from three or more independent experiments. n.s., non-significant; ****P ≤ 0.0001, **P ≤ 0.01, estimated by two-tailed unpaired Student's t-test.

Figure S5 (Related to Figure 6)

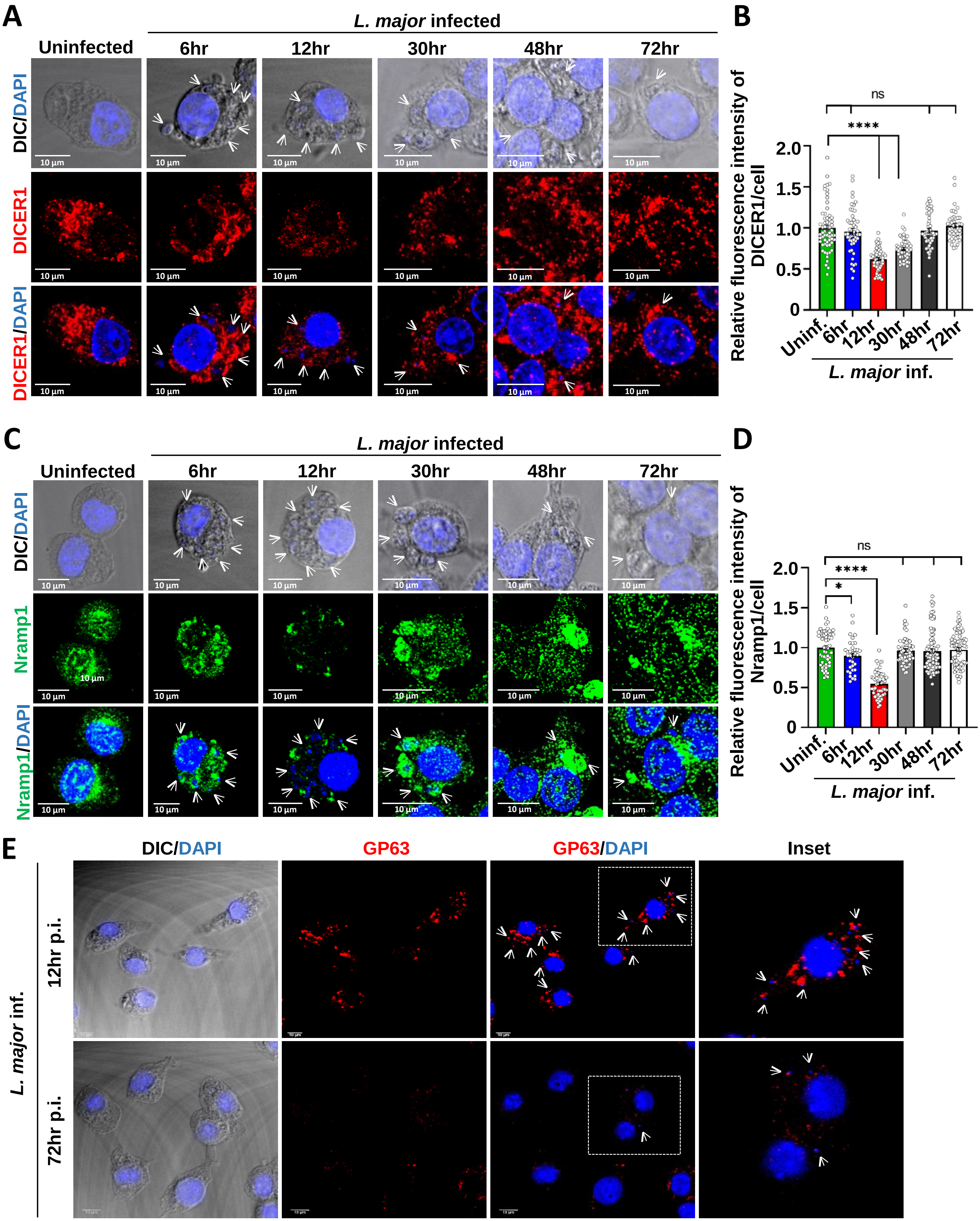

Figure S5. **Alteration of DICER1 and Nramp1 protein levels during the course of *L. major* infection in macrophage cells. (A)** Immunofluorescence staining of DICER1 (red) in uninfected or *L. major*-infected J774A.1 macrophages at different time points post-infection (6-72 hours). Nuclei were stained with DAPI (blue).

The DIC/DAPI panel indicates intracellular parasites (smaller nuclei, marked with white arrows). Images were acquired using a Leica SP8 confocal microscope with a 63× objective. **(B)** Quantification of the DICER1 fluorescence intensities in the respective images shown in bar diagram. Values are expressed as means ± SEMs (at least 44 cells from N = 3 independent experiments were analyzed). **(C)** Immunofluorescence staining of Nramp1 (green) in uninfected or *L. major*-infected J774A.1 macrophages at different time points post-infection (6-72 hours). Nuclei were stained with DAPI (blue), and intracellular parasites are indicated by white arrows. Images were acquired with a Leica SP8 confocal microscope, 63× objective. **(D)** Quantification of the Nramp1 fluorescence intensities in the respective images shown in bar diagram. Values are expressed as means ± SEMs (at least 35 cells from N = 3 independent experiments were analyzed). **(E)** Immunofluorescence staining of GP63 (red) in *L. major*-infected J774A.1 macrophages at 12 hours and 72 hours post infection. Nuclei were stained with DAPI (blue), and intracellular parasites are marked with white arrows. In the bar diagram, individual values are shown as small circles. n.s., non-significant, estimated by two-tailed unpaired Student's t-test or one-way ANOVA.

Figure S6 (Related to Figure 7)

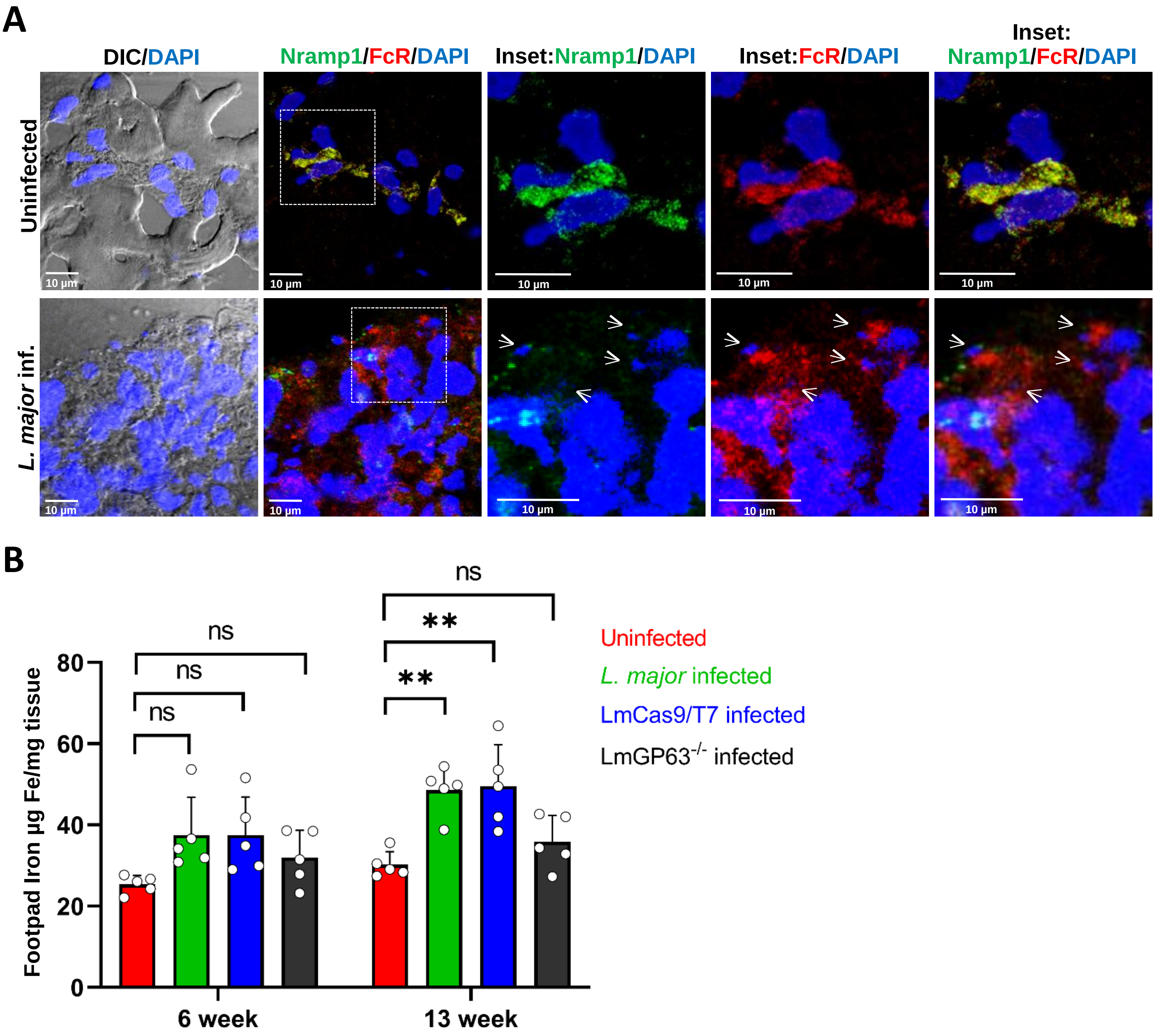

Figure S6. **Analysis of mouse footpads infected with wild type *L. major*, LmCas9/T7, or LmGP63$^{-/-}$ strains. (A)** Immunofluorescence staining for Nramp1 (green) and the macrophage-specific marker CD32 (FcR) (red) in the footpad cryosections of BALB/c mouse that were either uninfected or infected with wild type *L. major*. Tissues were harvested at 6 weeks post infection (p.i.). Nuclei were stained with DAPI (blue) and small *Leishmania* nuclei are marked with white arrows. The insets are zoomed regions marked by boxes showing co-localization of Nramp1 and CD32 (FcR). Images were acquired with Leica SP8 confocal, 63× objective. **(B)** Bar diagram showing iron content in mouse footpad tissues at 6 and 13 weeks p.i. Footpad tissues from uninfected mice or those infected with *L. major*, LmCas9/T7, or LmGP63$^{-/-}$ strains were excised and processed for ferrozine-based iron quantification. Data are represented as mean iron content for uninfected (red bar), *L. major* (green), LmCas9/T7 (blue), and LmGP63$^{-/-}$ (black). Each group included five mice (n = 5), and SEM values were calculated from three independent experiments. n.s., non-significant; **P ≤ 0.01 estimated by two-tailed unpaired Student's t-test.

Table S1. List of reagents used in the study

**Reagent**	**Source**	**Identifier**
Adenine	Sigma-Aldrich	Cat# A2786
Agarose	SRL	Cat#36601
Ammonium acetate	Merck	Cat#101116
Blasticidin S HCL	Gibco	Cat#R21001
BSA	Sigma-Aldrich	Cat#A2153
CaCl2	Sigma-Aldrich	Cat#C5080
DMEM	Gibco	Cat#12100061
EDTA	G-Biosciences	Cat#RC047
FeCl3	Sigma-Aldrich	Cat#701122
Ferrozine	Sigma-Aldrich	Cat#160601
FBS	Gibco	Cat#97068-085
Folic acid	Sigma-Aldrich	Cat#F8758
Gentamicin	Abbott	N/A
Gelatin	AMRESCO	Cat#9764
Glutaraldehyde	Sigma-Aldrich	Cat#G7776
HEPES	Sigma-Aldrich	Cat#3784
Hemin	Sigma-Aldrich	Cat# 51280
HCL	Merck	Cat#1930010521
KMnO4	Merck	Cat#105082
L-Ascorbic acid	Sigma-Aldrich	Cat#A92902
Lipofectamine 2000 tranfection reagent	Invitrogen	Cat#11668019
M199	Gibco	Cat#31100019
NaCl	Merck	Cat# 106404
NaHCO3	Sigma-Aldrich	Cat#S5761
Neocuproine	Sigma-Aldrich	Cat#N1501
Osmium tetroxide	Sigma-Aldrich	Cat#75632
Optimal cutting temperature (OCT) compound	Tissue-Tek	Cat#4583

Penicillin-streptomycin	Gibco	Cat#10378016
Puromycin Dihydrochloride	Merck	Cat#P8833
poly-L-lysin	Sigma-Aldrich	Cat#P4707
polybrene	Sigma-Aldrich	Cat#TR-1003
PMSF	Sigma-Aldrich	Cat# P7626
PVDF	Millipore	Cat#IPVH00010
Sodium pyruvate	Sigma-Aldrich	Cat#P5280
SIGMAFAST Protease Inhibitor Cocktail Tablet, EDTA-free	Sigma-Aldrich	Cat#S8830
SDS	Sigma-Aldrich	Cat#L4390
Super Signal West Pico Chemiluminescent Substrate	Thermo Fisher Scientific	Cat#34580
SYBR green fluorophore	BioRad	Cat#: 1725121
Sucrose	Merck	Cat#1.94953.0521
Skim Milk Powder	Millipore	Cat#70166
Tween 20	Sigma-Aldrich	Cat#93773
Tris	Sigma-Aldrich	Cat#T6066
TritonX -100	Sigma-Aldrich	Cat#T9284
Trypsin	Gibco	Cat#25200056
TRIzol reagent	Invitrogen	Cat#15596026
Vectashield	Vecta laboratories	Cat#H1000
Zncl2	Merck	Cat#108816
1,10-Phen	Sigma-Aldrich	Cat#131377
Heparin	Sigma-Aldrich	Cat#H3149
0.22um Syringe filters	AXIVA	Cat#SFPS33R
Total Exosome Isolation Reagent	Invitrogen	Cat# 4478359
Verso cDNA synthesis kit	Thermo Fisher Scientific	Cat# AB1453A
PureLink Quick Plasmid Miniprep Kit	Invitrogen	Cat# K210010
DNase1	Thermo Fisher Scientific	Cat# 18068-015
Phusion enzyme	Thermo Fisher Scientific	Cat# F530S

Table S2. List of antibodies used in the study

Antibody	Source	Identifier
Rabbit anti-Nramp1	IMGENEX India custom antibody generation facility	N/A
Rabbit anti-Hepcidin	Dr. William S. Sly (Saint Louis University School of Medicine)	N/A
Rabbit anti-Actin	Biobharati	Cat#BB-AB0025<br>RRID: Not available
Rabbit anti-*L. donovani* Actin	Dr. Amogh Anant Sahasrabuddhe (CSIR-Central Drug Research Institute)	N/A
Rabbit anti-Tubulin	Biobharati	Cat#BB-AB0118<br>RRID: Not available
Rabbit anti-*L. maxicana*- PGAM	Dr. Frederic Bringaud (University of Bordeaux)	N/A
Mouse anti- *Leishmania* gp63	Invitrogen	Cat#MA1-81830<br>RRID: AB_934457
Mouse anti- Ubiquitin	CST	Cat#3936<br>RRID: AB_331292
Mouse anti- DICER1	Antibody.com	Cat#A304979<br>RRID: Not available
Goat Anti-Rabbit IgG-Peroxidase	Sigma Aldrich	Cat#A9169<br>RRID: AB_258434
Rabbit Anti-Mouse IgG-Peroxidase	Sigma Aldrich	Cat#A9044<br>RRID: AB_258431
Goat anti-rabbit Alexa Fluor 488	Invitrogen	Cat#A11034,<br>RRID: AB_2576217
Goat anti-mouse Alexa Fluor 568	Invitrogen	Cat#A11031<br>RRID: AB_144696

Table S3. List of Oligonucleotides / primers and plasmids used in the study

**Oligonucleotides / primers**	**Source**
β-actin real-time FP-<br>TTACTCACTCGGACCAGCAC	IDT
β-actin real-time RP-<br>GGGGGCTCTTGTCACTAATCAT	IDT
Nramp1 real-time FP-<br>TGTCTCCTGCTTCTCCTCCT	IDT
Nramp1 real-time RP-<br>CTCTGTAGTCTGTCTCAT	IDT
Hepcidin real-time FP-<br>GGCTGTATTCCCCTCCATCG	IDT
Hepcidin real-time RP-<br>CCAGTTGGTAACAATGCCATGT	IDT
Pre-miRNA real-time 122 FP-<br>CCTTAGCAGAGCTGTGGAG	IDT
Pre-miRNA real-time 122 RP-<br>GCCTAGCAGTAGCTATTTAG	IDT
GP63-1 5’UTR<br>CCCCTTCTCGGTCATCCCACCGTTTCACCGGTATAATGCAGACC<br>TGCTGC	IDT
GP63-1 3’UTR<br>CCCATGGGATAGGGAGAAGCATGCCCACCGCCAATTTGAGAGA<br>CCTGTGC	IDT
5’ GP63sgRNA-<br>GAAATTAATACGACTCACTATAGGCTGCGATGTGGAAGAGAAG<br>TGTTTTAGACTAGAAATAGC	IDT
3’ GP63sgRNA-<br>GAAATTAATACGACTCACTATAGGTACCGAATCCGGCCGCTAC<br>GGTTTTAGAGCTAGAAATAGC	IDT
G00-	IDT

AAAAGCACCGACTCGGTGCCACTTTTTCAAGTTGATAACGGAC TAGCCTTATTTTAACTTGCTATTTCTAGCTCTAAAAC	
Cas9 FP- GACAAGAAGTACAGCATCGGCCTG	IDT
Ca9 RP- GTCGCCTCCCAGCTGAGA	IDT
T7 pol FP- ATGGAGACGATTAACATCGCTAAGAAC	IDT
T7 pol RP- CTACGCGAACGCGAAGTC	IDT
rRNA45 FP- CCTACCATGCCGTGTCCTTCTA	IDT
rRNA45 RP- AACGACCCCTGCAGCAATAC	IDT
P1- ATGACTGAATACAAGCCAACGGT	IDT
P2- TTAGGCTCCCGGCTTACG	IDT
P3- ATGCCTTTGTCTCAAGAAGAATCC	IDT
P4- GCCCTCCCACACATAACC	IDT
P5- CTCCCGAGCGCCATTCTACAC	IDT
P6- CTAGAGCGCCACGGCCA	IDT
**Plasmids**	
pTB007, pTBLAST and pTPURO	Gift from Dr. Subrata Adak
